# Photorespiration, Drought and the Assimilation–Growth Gap: Bounding the Forgone Wood in High-Elevation *Cedrus libani* and *Juniperus excelsa* Forests of the Eastern Mediterranean

**DOI:** 10.3390/plants15213441

**Published:** 2026-11-05

**Authors:** Emre Yazar, Bülent Akgün, Emre Babur, Sinan Kordon, Erdi Ekren

**Affiliations:** 1 Graduate Education Institute, Department of Bioengineering and Sciences, Kahramanmaraş Sütçü İmam University, Kahramanmaraş 46050, Türkiye; emre.yazar6@gmail.com; 2 Department of Forest Engineering, Faculty of Forestry, Kahramanmaraş Sütçü İmam University, Kahramanmaraş 46050, Türkiye; 3 Department of Landscape Architecture, Faculty of Forestry, Kahramanmaraş Sütçü İmam University, Kahramanmaraş 46050, Türkiye; sinankordon@ksu.edu.tr (S.K.); eekren@ksu.edu.tr (E.E.)

**Keywords:** *Cedrus libani*, *Juniperus excelsa*, photorespiration, drought stress, anisohydry, source–sink limitation, carbon sequestration

## Abstract

Türkiye holds the world’s largest natural stands of Lebanon cedar (*Cedrus libani* A. Rich.) and extensive Crimean juniper forest (*Juniperus excelsa* M. Bieb.), together comprising 884,361 ha of closed, drought-exposed forest. Both assimilate at needle water potentials near −3.0 MPa, yet lose part of their potential assimilation to photorespiration and to stomatal closure under water deficit. A literature-parameterized scenario model relaxes both constraints against a radiation-limited ceiling. The zonal photorespiratory and stomatal input fractions are structured expert estimates, not measurements, which are: 8–16% and 14–28% of potential gross primary production (GPP), or 13–23% and 11–23% after coupling. Together they reduce weighted-mean net primary productivity from 3.29 to 2.17 Mg C ha^−1^ yr^−1^, a 34.1% reduction in potential GPP (Monte Carlo interval 31.0–36.4%, conditional on the elicitation). Of that loss, 39–46% is obligate photorespiration beyond management reach, depending on how the jointly caused interaction is assigned; the obligate share falls along the aridity gradient. Radial growth in these species occupies a narrow climatic window that does not coincide with peak assimilation, so forgone wood is reported as a function of the source-control coefficient *φ*, with *φ* = 1 as an upper bound. At *φ* = 1, the estate forgoes up to 1.5 million m^3^ yr^−1^ of stemwood, and the 3.6 million Mg yr^−1^ of unfixed carbon (as CO_2_-equivalent, not net sequestration) is worth, together with that wood, about US$ 0.20 billion; at *φ* = 0.5 the wood and the value halve, and at *φ* = 0 no wood is forgone.

## 1. Introduction

Türkiye’s forest estate covers 23,375,331 ha, or 29.97% of the country’s 78,004,644 ha land surface, and holds a carbon stock of about 2.1 billion Mg [1]. It sustains both the rural economy and a substantial share of the national sink, but 41% of it is classified as sparsely rather than normally closed—steep, shallow-soiled, karstic uplands unsuitable for commercial pines [1,2]. In these difficult environments, two long-lived conifers play a key role. Lebanon or Taurus cedar (*Cedrus libani* A. Rich.) occupies 644,149 ha, of which 505,413 ha are normally closed stands, and represents more than half of the species’ global distribution [1,3,4]. Juniper forest, dominated by Crimean juniper (*Juniperus excelsa* M. Bieb.), is the fourth-most extensive forest type in the country at 1,849,526 ha, but only 378,948 ha of that is normally closed; the remaining 1.47 million ha is open, sparse woodland across the supra- and oro-Mediterranean belts and the forest-steppe of the interior [1,2].

Both species are recognized as stress specialists, exhibiting frost hardiness, drought tolerance, and anisohydric behavior. These physiological adaptations allow them to establish in environments unsuitable for other species. Consequently, their carbon economy is governed by constraints distinct from those of lowland pines, a balance that has yet to be quantified.

Two processes are jointly responsible for the shortfall between potential and realized assimilation in any C_3_ conifer. The first is photorespiration, the unavoidable side-reaction in which Rubisco fixes O_2_ rather than CO_2_, releasing previously assimilated carbon and consuming ATP and NADPH in the recycling pathway [5,6,7]. The second is stomatal closure under water deficit, which protects the hydraulic system but curtails CO_2_ supply to the mesophyll and depresses net assimilation [8,9]. The two are coupled: a stomatal-driven fall in intercellular CO_2_ concentration simultaneously favors the oxygenase reaction, so stomatal limitation carries an additional photorespiratory penalty within the same physiological window [10,11]. In well-watered C_3_ foliage, the photorespiratory release reaches about 26% of the rate of net CO_2_ assimilation at 25 °C and 350 µmol mol^−1^, equivalent to roughly a fifth of gross assimilation, and climbs steeply above the thermal optimum [7]. Direct gas-exchange work confirms that drought and heat interact strongly in both genera: in *Cedrus* seedlings drought depresses photosynthesis largely through stomatal closure, while raising rather than lowering the thermotolerance of photosystem II [12], and controlled soil- and air-drought experiments show pronounced declines in leaf gas exchange and hydraulic conductivity as water potential falls, with cedar maintaining assimilation to lower potentials than most co-occurring species but at a rising photorespiratory and diffusional cost [13,14].

Stomatal closure is the proximate driver of carbon loss under drought in both species, although in this model much of the carbon it costs is released through the photorespiratory route, which it amplifies, rather than through diffusive limitation alone. As soil water declines and vapor pressure deficit rises, guard cells lose turgor in an ABA-mediated response, lowering stomatal conductance and curtailing CO_2_ diffusion to the mesophyll [8,9]. Because cedar and juniper are anisohydric, they allow water potential to fall to about −3.0 MPa or below and sustain gas exchange deep into drought through osmotic adjustment [13,15]. This persistence is adaptive for survival, but it extends the window over which the coupled stomatal-plus-photorespiratory tax operates and raises the risk of hydraulic failure under the most extreme deficits [16]. Two years of hourly dendrometer monitoring of co-occurring cedar and juniper in the Taurus showed both species at their highest daily stem shrinkage in July and August, with cedar recovering from water-deficit episodes faster than juniper [15].

For cedar and juniper, the balance between these two terms is expected to differ from that of coastal pines. Their elevation moderates the photorespiratory penalty because mean growing-season temperatures at 1200–2200 m remain below those of the coastal belt, but the aridity of their range intensifies the stomatal term. Whether this trade-off leaves them physiologically constrained overall remains an open question, with practical consequences for afforestation planning on degraded uplands.

What has not been established is the size of that constraint in the terms forest policy actually uses. Carbon accounting for the Turkish estate rests on remote-sensing and inventory products—MODIS-derived NPP evaluated against national field plots [17], ecosystem carbon budgets for Mediterranean conifers [18], and, more recently, process-based projections for *Pinus brutia* [19]—all of which report the carbon these forests did fix, not the carbon they were physiologically prevented from fixing. The economic literature has the same shape: it values the standing sink and its trading potential [20,21] without attributing the shortfall to a mechanism. No study has partitioned the gap between potential and realized assimilation in Turkish cedar and juniper into its photorespiratory and stomatal components. This omission matters because the national forest estate is counted on to offset roughly 11% of Türkiye’s greenhouse-gas emissions on the path to the 2053 net-zero target [22], and a sink already operating well below its radiation-limited ceiling will respond to management differently from one that is not.

Carrying such a partition through to wood raises a second question, and it is the one on which the usefulness of any source-side carbon accounting turns. Models of this kind convert forgone assimilation into forgone stem volume using a fixed allocation fraction, assuming wood production tracks carbon supply. The evidence for that presumption is weak and contested. Across 78 eddy-covariance sites, the correlation between gross primary production and radial growth reaches only r = 0.26 at the 31 sites with on-site tree-ring data, versus r ≈ 0.7–0.9 under strong source control, and decoupling is greater where it is drier [23]. Mechanistically, turgor rather than carbon confines radial growth over much of the water-potential range in which conifers photosynthesize [24,25], and hourly dendrometry in the Taurus shows that the radial growth of both species is restricted to a narrow climatic window that does not coincide with the hours or the months of peak assimilation [15]. The interpretation is not settled—Trugman and Anderegg [26] argue that there is no direct evidence for widespread organismal sink limitation and that source-limited models still reproduce observed productivity patterns—and this study has no data with which to settle it. What it can do is refuse to assume the answer.

This study confronts the identified knowledge gap by synthesizing published gas-exchange, dendrometer, and dendroclimatic data to generate zone-specific estimates of the fraction of potential GPP lost to each mechanism across the cedar and juniper ranges in Türkiye. A counterfactual model is developed in which both physiological constraints are computationally relaxed. Relative to the companion study of the lowland pines *Pinus brutia* and *P. nigra* [27], which established the modeling framework, this study extends it in three ways. It applies the framework to two anisohydric, high-elevation species in which the stomatal rather than the photorespiratory term is expected to dominate; it partitions the loss into an obligate tier that no management can remove and a drought-attributable tier; and it treats the step from forgone assimilation to forgone wood as conditional on source control rather than fixed. We report the partition of assimilation loss as the primary result because it does not depend on what becomes of the carbon. The translation into stem volume and monetary value is reported second and conditionally, as a function of how much of a source-side gain would be realized as wood, rather than at the single value that fixed allocation implies. The counterfactual scenario is not biologically attainable and serves solely as an analytical benchmark, establishing an upper limit on what adaptive management interventions could potentially recover, rather than a management target.

## 2. Results

### 2.1. Decomposition of the Weighted-Mean Carbon Flow

**Figure 1:**
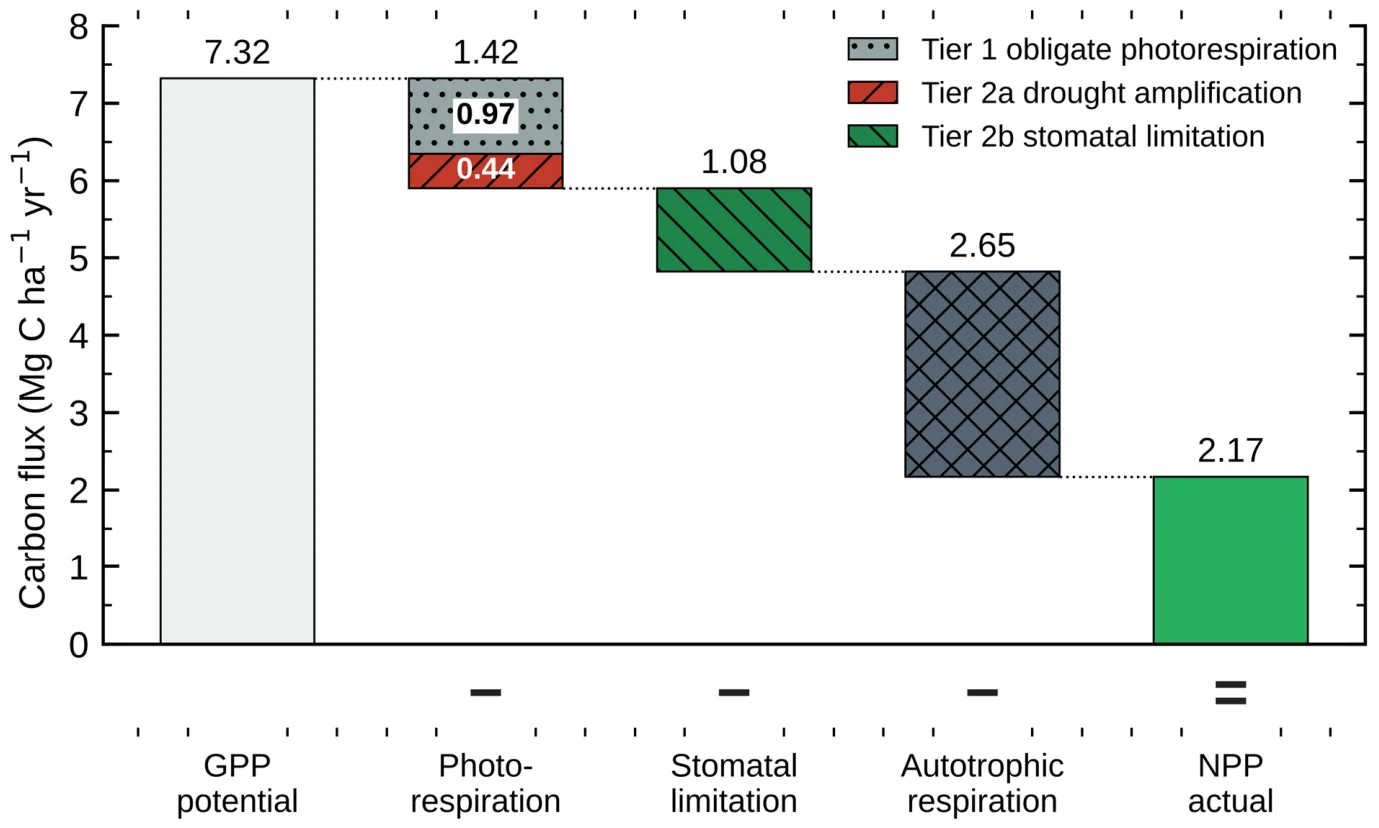
Waterfall decomposition of the weighted-mean carbon flow from potential GPP to actual NPP across the combined closed-canopy *C. libani* and *J. excelsa* estate in Türkiye. Photorespiration and stomatal limitation jointly remove 2.50 Mg C ha^−1^ yr^−1^, or 34.1% of potential GPP, of which 39% is obligate photorespiration and 61% is drought-attributable.

Weighting each zone × species combination by its area, the potential-GPP proxy (APAR × RUE_pot_) for the combined 884,361 ha closed-canopy estate is 7.32 Mg C ha^−1^ yr^−1^ (Figure 1). Photorespiration removes 1.42 Mg C; stomatal limitation removes a further 1.08 Mg C; autotrophic respiration absorbs 2.65 Mg C; what remains as actual NPP is 2.17 Mg C ha^−1^ yr^−1^. The two physiological constraints targeted here jointly remove 2.50 Mg C ha^−1^ yr^−1^, equivalent to 34.1% of potential GPP. Because carbon-use efficiency enters both terms as a constant, the same figure follows identically from the ratio of actual to counterfactual NPP (2.17 against 3.29 Mg C ha^−1^ yr^−1^); the two routes agree by construction and the agreement is reported as a check on the aggregation arithmetic, not as independent evidence. The realized state implied by the model is compared with independent benchmarks in Section 3.2. The stomatal share of that loss is proportionally larger than in the coastal pines, consistent with the arid, high-elevation character of the cedar–juniper estate.

### 2.2. Obligate and Drought-Attributable Components of the Loss

Not all of this loss is of the same kind, and the distinction matters more than the total. Photorespiration in a well-watered C_3_ leaf is an obligate consequence of Rubisco’s dual specificity: it is a cost of the photosynthetic pathway itself; it is not a symptom of stress, and no silvicultural intervention can remove it. Stomatal closure under water deficit is different in kind again—it is a protective response that maintains hydraulic integrity, so the carbon it forgoes is the price of survival rather than an avoidable inefficiency. Following the companion study [27], we therefore partition the combined loss into three additive tiers: Tier 1, obligate photorespiration at well-watered conditions (*f*_PR_ applied to potential GPP); Tier 2a, the drought amplification of the photorespiratory term through the diffusive and thermal routes (*f*_PR_ *λ f*_ST_); and Tier 2b, stomatal limitation itself. The three sum exactly to the combined loss. The partition is not unique, because Tier 2a is jointly caused: removing drought altogether leaves a photorespiratory loss of 39.0% of the total, removing photorespiration altogether leaves a stomatal loss of 53.8%, and the remaining 7.3% is the interaction between them. The main effects here are the losses computed with the other fraction set to zero; Tier 2a (17.7% of the loss) is the drought amplification of photorespiration, not this interaction term. We assign that interaction entirely to the drought-attributable component, which is the convention that maximizes the share open to management; splitting it equally between the two main effects would put the obligate share at 43% rather than 39% and assign it wholly to photorespiration at 46%. All three conventions are reported as results (obligate share 39%, 43% or 46%; drought-attributable 61%, 57% or 54%), and every obligate/drought figure below depends on this choice.

**Figure 2:**
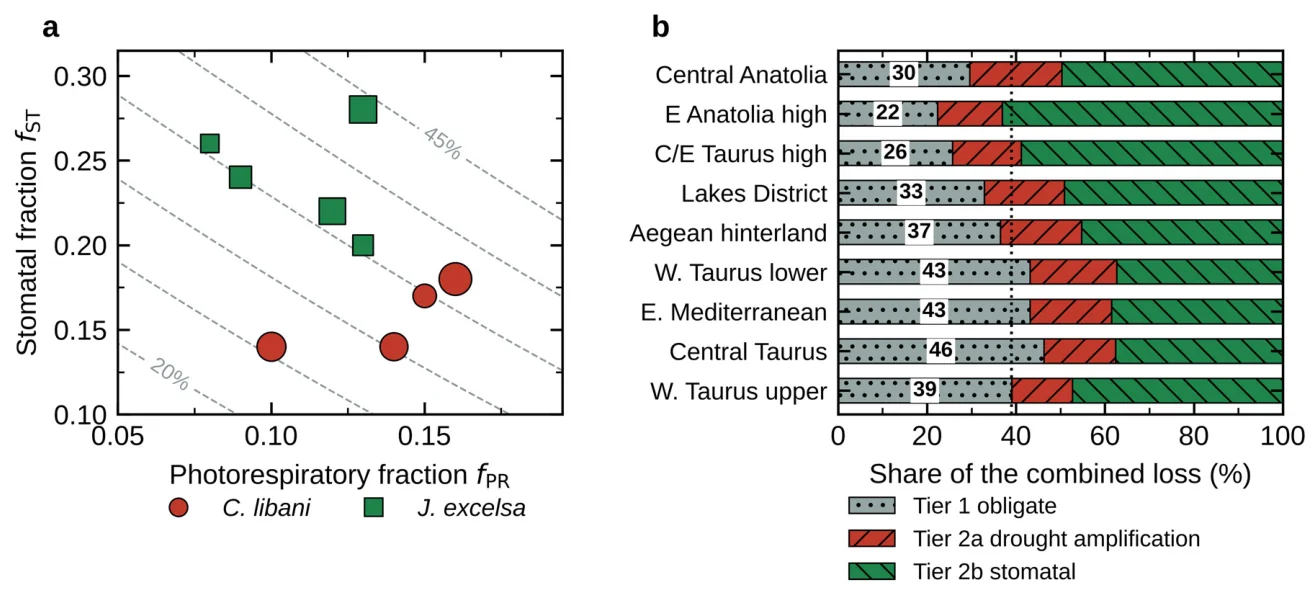
Structure of the modeled stress fractions. (**a**) The nine zone × species units in photorespiratory (*f*_PR_) and stomatal (*f*_ST_) space, with symbol area scaled to zone area and dashed iso-lines of the combined reduction 1 − (1 − *f*_PR_(1 + *λ* fST))(1 − fST) at 5% intervals; cedar zones occupy the warm, moist corner and juniper zones the cool, dry one, and all but two sit between the 30% and 40% iso-lines, the exceptions being W. Taurus upper at 26% and the Central Anatolian steppe at 44%. (**b**) The same units ordered by stomatal fraction, with the loss partitioned into the three tiers of Section 2.2. The dotted line marks the estate-wide obligate share of 39%. All values are model inputs and outputs listed in Section 4.1 and Section 4.2; nothing in this figure is interpolated or schematic.

Across the combined estate, Tier 1 accounts for 0.97 Mg C ha^−1^ yr^−1^ (13.3% of potential GPP), Tier 2a for 0.44 Mg C ha^−1^ yr^−1^ (6.1%), and Tier 2b for 1.08 Mg C ha^−1^ yr^−1^ (14.8%). Obligate photorespiration is therefore 39% of the total loss and the drought-attributable component (Tiers 2a and 2b together) is 61%. Only the latter could be addressed by management in any sense, and it corresponds to 0.61 Mt C yr^−1^, or 2.23 Mt CO_2_ yr^−1^, against the headline figure of 3.65 Mt CO_2_ yr^−1^; it is a ceiling on what management could address, not an estimate of what it would recover. The obligate share varies systematically along the aridity gradient (Figure 2b), falling from 39–46% in the cedar zones to 22–30% in the driest juniper zones of Central and Eastern Anatolia: the drier the site, the larger the fraction of the loss that is drought-attributable rather than intrinsic. The obligate share is a function of the zone’s pair (*f*_PR_, *f*_ST_) and *λ* alone; across the nine units it tracks the ratio *f*_PR_/*f*_ST_ (r = 0.99). The gradient therefore reflects where the summer-stress index places each zone rather than species identity: the Aegean hinterland juniper unit (ratio 0.65) sits beside the upper W. Taurus cedar unit (0.71) at 37–39%, while the three driest juniper units (Central Anatolia, Eastern Anatolia and the high Central/Eastern Taurus), with ratios of 0.31–0.46, fall to 22–30%. Throughout this paper, the word “deficit” denotes this shortfall measured against the counterfactual benchmark, and not a recoverable sum. Of the headline quantities reported below, only the drought-attributable 61% could be addressed by management to any degree; the obligate tier should be read as a baseline cost of C_3_ physiology rather than as forgone value. Where a potentially recoverable quantity is intended, it is named as the drought-attributable component and reported separately.

### 2.3. Aggregate Carbon and Stem-Volume Deficits

Scaling the zonal output across the estate and taking *φ* = 1 so that allocation proceeds as observed—an upper bound rather than a central value (Section 2.7)—yields an annual forgone stem volume of ~1.5 million m^3^ yr^−1^ and an annual forgone CO_2_-equivalent carbon of ~3.6 million Mg CO_2_ yr^−1^ (Table 1). Cedar contributes most of the forgone volume, since its wood is both more productive and far more valuable. Junipers contribute disproportionately to the forgone-carbon term relative to their volume, reflecting their role as low-value but wide-ranging carbon stewards of the arid interior. On the closed-canopy basis adopted here, their share of the estate is far smaller than their share of the national juniper area, four-fifths of which is open woodland lying outside the scope of this model. Because juniper’s stem allocation and wood density are assumptions rather than measurements (Section 4.4), its volume and value entries in Table 1 are indicative, and their two-decimal precision reflects the arithmetic rather than the inputs.

### 2.4. Sensitivity Analysis

**Figure 3:**
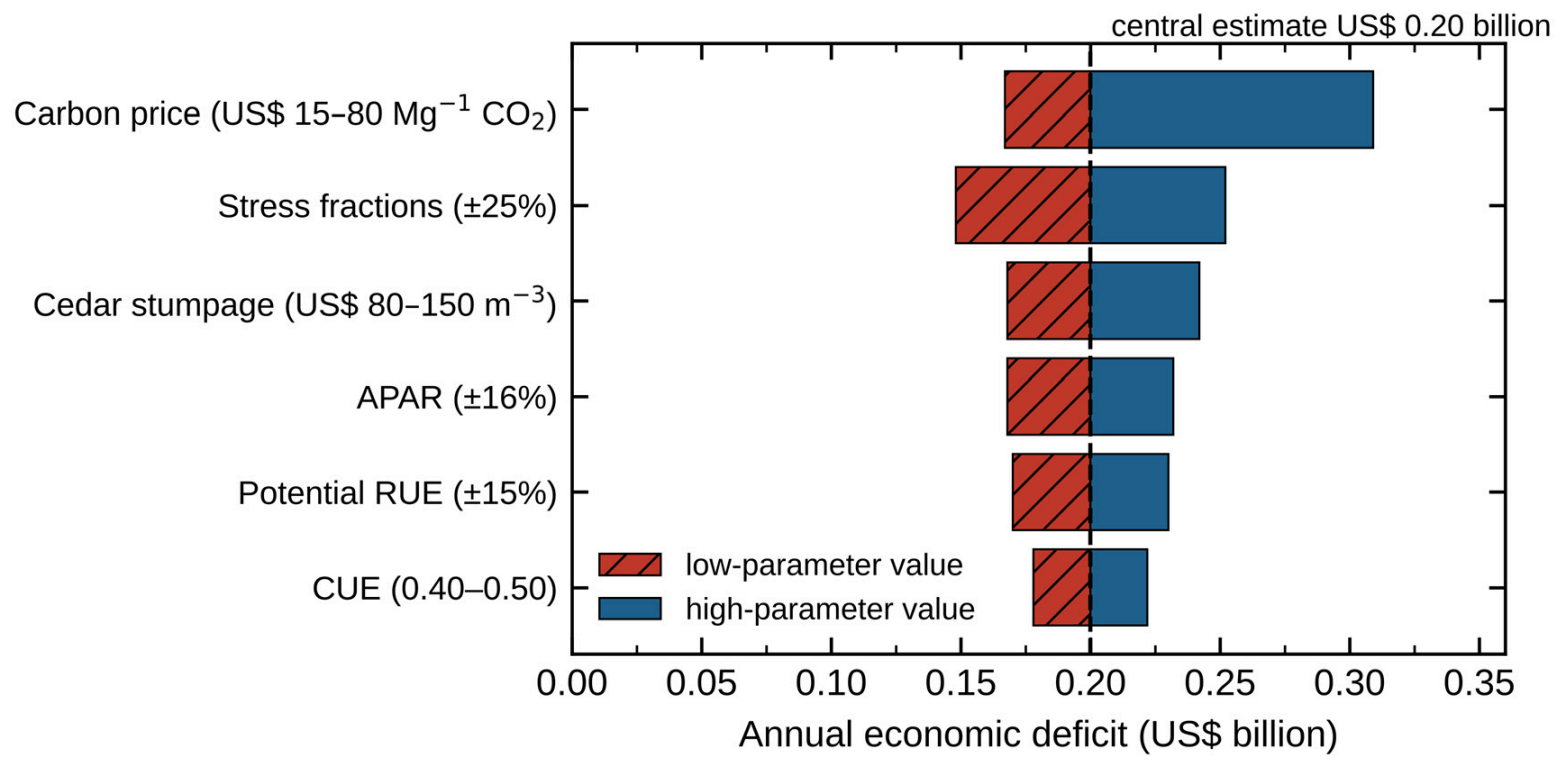
Tornado sensitivity of the annual deficit around the central estimate. The stress fractions are the most influential biophysical input, with absorbed radiation and potential RUE close behind, and the carbon price the most influential economic one. The bars span the range of magnitudes across the tested parameter ranges; the positive sign of the deficit is a property of the model structure, not a result.

The one-at-a-time tornado analysis (Figure 3) identifies stress fractions as the most influential biophysical inputs, with absorbed radiation and potential RUE close behind, and carbon price as the most influential economic input, followed by cedar stumpage. Across the envelope tested, the annual deficit spans about US$ 0.15–0.31 billion. The sign of the deficit is not a finding of this analysis: the model returns a positive loss for any positive stress fraction, so the tornado plot should be read for the range of magnitudes it spans, not as evidence that a loss exists. The coupling coefficient was examined separately as a bounding case: setting *λ* = 0, which removes the photorespiration–stomatal interaction altogether, lowers the combined reduction from 34.1% to 29.2% of potential GPP, and over the derived envelope of *λ* (1.45–2.85, Section 4.2) the combined reduction moves from 32.1% to 34.8% and the obligate share moves from 41.5% to 38.2%. The interaction therefore accounts for about five percentage points of the total at *λ* = 2.5 (2.8–5.6 points across the envelope); the headline moves by −2.1 to +0.7 points within the envelope, and the central conclusion is not an artifact of the coupling term.

### 2.5. Monte Carlo Uncertainty Intervals

**Figure 4:**
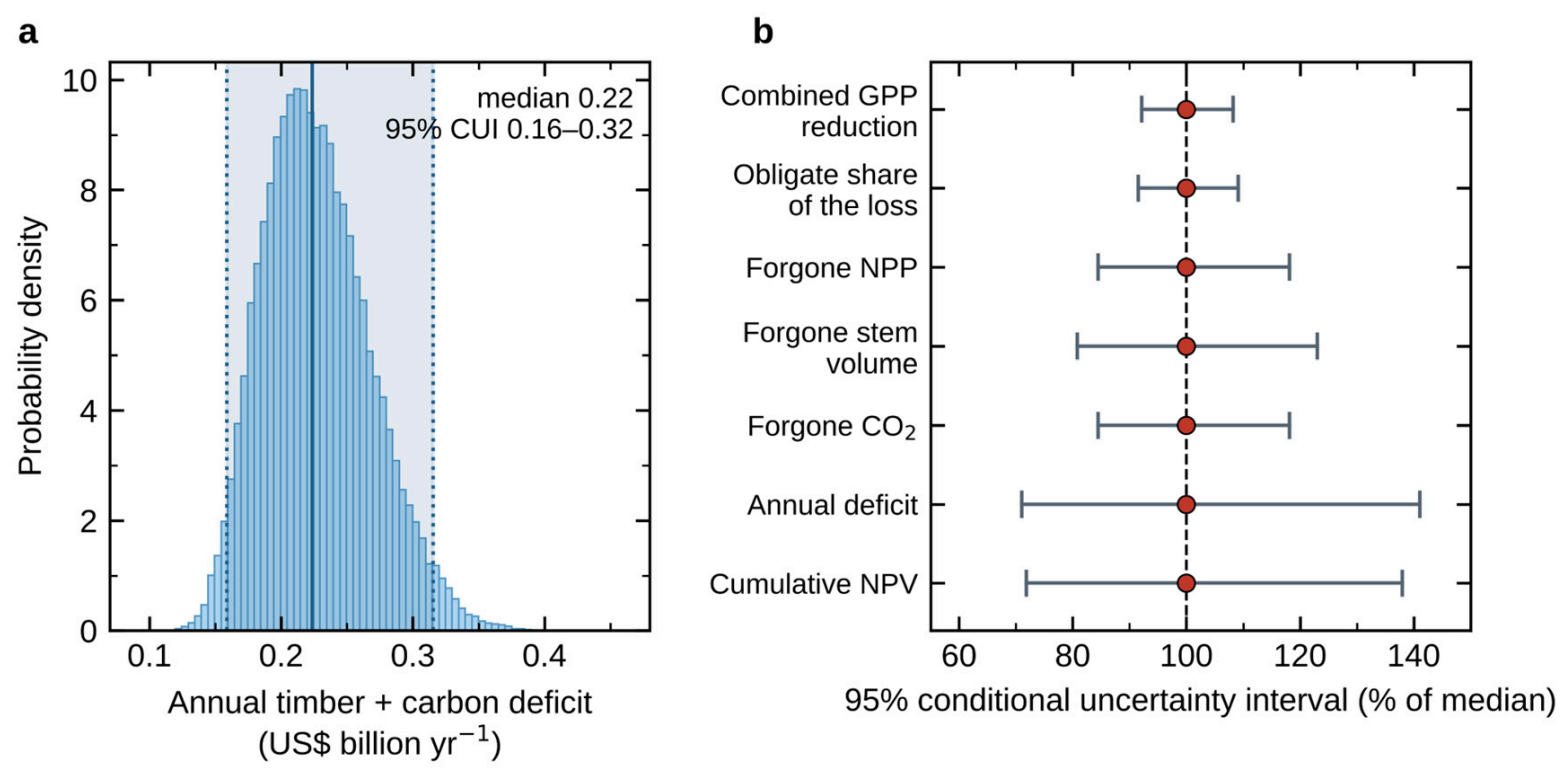
Monte Carlo uncertainty analysis (N = 40,000). (**a**) Probability density of the annual deficit, with the median (solid line) and 95% conditional uncertainty interval (dotted lines, shaded band). (**b**) Forest plot of the 95% conditional uncertainty intervals for the principal outputs, each expressed as a percentage of its own median (dot); the dashed line marks the median.

The Monte Carlo intervals show how far the outputs move when the inputs are varied jointly over their assigned ranges (Table 2, Figure 4; distributions in Section 4.6). They are conditional on those ranges being centered correctly and therefore measure the spread of the elicitation, not the accuracy of the result; for that reason they are reported as 95% conditional uncertainty intervals (CUI, the 2.5th–97.5th percentiles of the draws) rather than as confidence intervals. The combined physiological suppression of potential GPP has a median of 33.6% (95% CUI 31.0–36.4%), and the forgone flows are tightly bound, with 0.98 (0.82–1.15) million Mg C yr^−1^ of net primary production, 1.43 (1.16–1.76) million m^3^ yr^−1^ of stem volume, and 3.58 (3.03–4.23) million Mg CO_2_ yr^−1^. Relative uncertainty is modest and comparable across the physical quantities, encompassing roughly 15–25% of the median (Figure 4b). The obligate share of the loss is itself well constrained, at 39.5% (95% CUI 36.1–43.1%), so the partition of Section 2.2 is insensitive to *λ* within its derived envelope. The economic aggregate is the most dispersed because the carbon price range is both wide and right-skewed. Zone weights were held fixed in these draws because the species totals are set by the inventory. A supplementary run that perturbed the relative weights of the nine units by a multiplicative factor with a 20% standard deviation and renormalized them to the inventory totals changed no median and no interval bound in Table 2 by more than two units in the last reported digit (Table S2).

### 2.6. Economic Scale of the Deficit

At the prices in Section 4.5 and at *φ* = 1, the forgone volume and sequestration together amount to approximately US$ 0.20 billion annually—US$ 0.13 billion of forgone stumpage and US$ 0.07 billion of forgone carbon outside the stem—with a Monte Carlo median of US$ 0.22 billion (95% CUI 0.16–0.32) and a cumulative 2000–2023 net present value of US$ 2.40 billion (1.72–3.31). Restricting the valuation to the drought-attributable component (Monte Carlo median 60.5%)—the only part that management could in principle address, and a ceiling on it rather than an estimate of what would be recovered—gives approximately US$ 0.12 billion annually, with a Monte Carlo median of US$ 0.14 billion (95% CUI 0.10–0.19) and a cumulative net present value of US$ 1.45 billion (1.04–2.01). These monetary values index the scale of the physiological deficit; because the dominant services of these forests are protective—watershed regulation and karst erosion control—rather than merchantable, they capture only part of what is at stake. They are not, however, a lower bound on the deficit itself: they are computed at φ = 1 and therefore upper-bound the forgone wood and its value, and they provide a partial, not full, account of what is at stake.

### 2.7. Dependence of the Wood and Value Results on Source Control

The assimilation results above are independent of what subsequently happens to the carbon. The wood and value results are not. Table 3 reports them across the full range of *φ*. At *φ* = 1, the estate forgoes 1.46 million m^3^ of stem volume and 3.65 million Mg of retained CO_2_ a year, worth US$ 0.20 billion; at *φ* = 0.5, half of that; and at *φ* = 0, none of it. The 34.1% reduction in potential GPP, the partition into 39% obligate and 61% drought-attributable, and the fall of the obligate share along the aridity gradient remain unchanged because they describe assimilation rather than allocation. No value of *φ* is adopted as a central case because none has been published for either species (Section 3.3); Table 3 is therefore a bound rather than a point estimate, and its intermediate rows allow a reader who adopts a value from that evidence to read off the corresponding wood and value.

## 3. Discussion

### 3.1. Validity of the Counterfactual and of Its Assumptions

The counterfactual relaxes two processes that cannot, in fact, be switched off, and the standing of every number above depends on how that device is read. It is not a forecast and not a recoverable sum. It is a bound: the carbon that these stands would have fixed had they been able to assimilate at their radiation-defined potential. Three questions decide whether that bound is informative. The first is whether the partition between obligate and drought-attributable loss is defensible; Section 2.2 shows that roughly two-fifths of the total (39–46%, depending on the convention) is obligate and therefore outside management reach, and only the remaining 54–61% has any policy meaning, as a ceiling on what management could address. The second is whether the stress fractions are plausible; they are structured expert estimates (Section 4.2), and the Monte Carlo envelope of Section 2.5 shows the proportional conclusion holds across the full range assigned to them, subject to the qualification set out below. The third is whether the carbon and economic chains are mutually consistent, which Section 3.2 addresses directly. Only after those three are settled does it make sense to ask what management could do, and the answer there is correspondingly modest.

Two properties of the construction qualify every number above and should be stated plainly. First, we do not separately identify potential radiation-use efficiency and the stress fractions from anything we observe. The model multiplies them together, and the only quantity available to anchor the product is realized NPP, so a lower stress fraction paired with a lower RUE_pot_ reproduces the same realized NPP and yields a smaller loss. The trade-off can be stated exactly: the estate-mean realized NPP of 2.17 Mg C ha^−1^ yr^−1^ is reproduced by every pair (RUE_pot_, *f*_combined_) satisfying NPP = APAR × RUE_pot_ × (1 − *f*_combined_) × CUE, so that RUE_pot_ values 20% and 10% below those adopted imply combined losses of 17.7% and 26.8% rather than 34.1%, and values 10% and 20% above imply 40.1% and 45.1%. The elicited fractions select one point on that curve. The 34.1% is therefore the arithmetic consequence of the elicited fractions rather than an estimate inferred from them, and the Monte Carlo interval of Section 2.5 describes the spread of that elicitation rather than the precision of a measurement. Second, RUE_pot_ is anchored on the MOD17 evergreen-needleleaf maximum, calibrated empirically against observed gross primary production and therefore already containing Rubisco oxygenation at ambient CO_2_ and O_2_, and defined with the temperature and vapor-pressure-deficit scalars at unity. Part of what the counterfactual subtracts has thus already been removed from the ceiling it subtracts from. Two biases follow, and they act in opposite directions. Because the ceiling already contains Rubisco oxygenation, it understates what a truly photorespiration-free ceiling would be, which makes the modeled loss too small. Because that same ceiling is evaluated with the temperature and vapor-pressure-deficit scalars at unity, it also strips cold limitation, growing-season length, and atmospheric drought out of the baseline; at 1400–2200 m those terms are substantial, and the shortfall they create is charged in this model to the photorespiratory and stomatal fractions, which makes the modeled loss too large. Neither bias can be measured here, and only the first can be bounded: a ceiling free of Rubisco oxygenation would exceed the MOD17 ceiling by roughly the photorespiratory fraction at ambient conditions, about a fifth of gross assimilation at 25 °C [7]. How far that understates the absolute loss depends on what is held fixed: with the elicited fractions held fixed, the loss scales with the ceiling and is understated by 20–25%; with the realized NPP of Section 3.2 held fixed, the whole addition to the ceiling becomes forgone assimilation and the loss is understated by roughly 60–70%. The second cannot be bounded without the daily temperature and vapor-pressure-deficit inputs of the MOD17 scalars for each zone; its sign is known and its magnitude is not, and the net sign of the two remains indeterminate. The absolute tonnages are therefore conditional on the specific MOD17 ceiling adopted and, with the fractions held fixed, scale linearly with it: a ceiling 20% lower or higher changes every absolute quantity in Table 1, Table 2 and Table 3 by 20% while leaving the proportional decomposition unchanged. That is a property of the construction rather than a caveat on it, and it is why the absolute tonnages should not be read as carbon that removing photorespiration would recover. The downward scaling of RUE_pot_ for the slow growth of these species works the other way, since that slow growth is itself partly a consequence of the water limitation the model goes on to charge through *f*_ST_. For this reason, we report the proportional decomposition as the less baseline-dependent result, and the absolute tonnages as quantities whose level has not been validated.

### 3.2. Internal Consistency of the Carbon and Economic Chain

The economic figures are a linear transformation of the carbon figures, so they add no independent evidence and cannot be more reliable than the carbon chain beneath them. That chain runs from a radiation-use-efficiency estimate of potential GPP, through the stress fractions, through carbon-use efficiency to NPP, and only then through stem allocation and wood density to merchantable volume. Each step loses information. The closest independent check available is the field carbon-budget estimate of about 1.6 Mg C ha^−1^ yr^−1^ reported for pure *C. libani* stands by Evrendilek et al. [18]. The modeled cedar NPP on the closed-canopy basis is 2.74 Mg C ha^−1^ yr^−1^. The two are not the same quantity—a net sequestration rate is NPP less heterotrophic respiration—so the comparison bounds the model from below rather than validating its level, and no measurement of NPP for these stands is available to test it against. A more directly checkable quantity is the realized stem increment the model implies, which follows from the same allocation and density parameters: 4.3 m^3^ ha^−1^ yr^−1^ for cedar and 1.7 m^3^ ha^−1^ yr^−1^ for juniper, against counterfactual values of 6.4 and 2.8. Two independent benchmarks are available for them. The 2025 national inventory reports a current annual increment of 48.1 million m^3^ on 13.37 million ha of normally closed high forest, 3.6 m^3^ ha^−1^ yr^−1^ averaged over all species [1]. The modeled realized increments bracket that mean, with cedar on productive karst sites above it and the slow-growing juniper below, which is as much as a species-aggregated statistic can show, since the inventory does not publish increment by species. The second is MODIS forest NPP evaluated against national field plots over 2008–2013 [17]: 1.5 Mg C ha^−1^ yr^−1^ for the Aegean–Mediterranean region and 2.1 for Central and Eastern Anatolia, with MODIS values about twice the field-derived values nationally. The modeled realized NPP of 2.56–2.83 Mg C ha^−1^ yr^−1^ for cedar is 1.7–1.9 times the Aegean–Mediterranean MODIS forest mean, whereas the 1.11–1.68 Mg C ha^−1^ yr^−1^ modeled for juniper lies within or below the regional means. If the national MODIS-to-field ratio holds regionally, the modeled cedar NPP is three to four times the field-derived regional forest mean, which aggregates all forest types and closure classes. The companion pine study found its modeled NPP to exceed forest-attributed MODIS values by roughly a factor of two [27]; the discrepancy here is of the same kind and in the same direction for cedar, and absolute tonnages should be read as upper estimates while the proportional decomposition, which does not depend on the baseline level, is the less baseline-dependent result. For the same reason, we report the drought-attributable fraction (2.23 Mt CO_2_ yr^−1^) rather than the headline total as the policy-relevant quantity.

### 3.3. Source and Sink Control of the Translation to Wood

The step from forgone assimilation to forgone wood is the weakest link in the chain, and it is weak for a reason that is specific to this system. Radial growth in conifers is limited by turgor, not by carbon supply, over much of the range in which these trees photosynthesize [28,29]. Growth probability in mature conifers falls to about 9% below a soil water potential of −0.6 MPa [24], and turgor-driven tracheid enlargement ceases below about −0.73 MPa in *Pinus sylvestris* at its xeric edge [25]. Both values are on a soil-water-potential basis—Cabon et al. [25] set cell water potential equal to soil water potential in the model from which theirs is fitted—and are therefore not directly comparable with the needle water potentials near −3.0 MPa to which cedar and juniper sustain gas exchange. A transpiring conifer with needles at −3.0 MPa at midday may stand on soil several megapascals less negative, so we do not rest the argument on that contrast. The evidence that does bear on it is phenological rather than thermodynamic, and it exists for these two species at a Taurus site. Over two years of hourly dendrometer monitoring in the Elmalı cedar research forest, radial growth in both species was confined to vapor pressure deficits below 1.9 kPa and to air temperatures between 0 and 20 °C, peaking at 11 °C, while the site itself reached 5.48 kPa and 36.3 °C; about 90% of juniper growth and 75% of cedar growth occurred below 0.24 kPa. Both species grew mainly between 02:00 and 07:00, and only 42% of juniper and 69% of cedar annual increment was laid down between April and October [15]. Over the same period, the maximum daily shrinkage of both species, which those authors read as a proxy for transpiration and assimilation, peaked in July and August. Growth and assimilation at this site are therefore largely out of phase, diurnally and seasonally. A source-side accounting that converts the one into the other at a fixed ratio will overstate the wood, and will overstate it most on the driest sites—which are precisely the sites where this study finds the drought-attributable share of the loss to be largest.

The cross-biome evidence points the same way. Correlations between eddy-covariance GPP and ring width reach only r = 0.26 at the 31 sites with on-site tree-ring data and r = 0.38 regionally, compared with the r ≈ 0.7–0.9 expected under strong source control, with about a tenth of observations in the source-limited range; the decoupling strengthens with aridity [23]. The mechanism is disputed even among those who find the decoupling. In a semi-arid *Juniperus monosperma*–*Pinus edulis* woodland, non-structural carbohydrates fell rather than accumulated as predawn water potential declined, which argues against the textbook picture in which growth stops first and the surplus is stored [30]. That study also cuts against the present argument in one respect, and we report it as such: growth did not cease before photosynthesis, and in *J. monosperma* the two converged and reached zero at a similar water potential—evidence for a narrower assimilation-without-growth window than the one invoked here, not a wider one. Trugman and Anderegg [26] go further and argue that no direct evidence exists for widespread organismal sink limitation, and that source-limited models continue to reproduce observed productivity patterns.

This study cannot adjudicate between those positions and does not try to. What matters here is that all three readings have the same consequence for the step this paper takes: under sink limitation, under co-limitation by water, and under any partial coupling, forgone assimilation does not map proportionally onto forgone wood. That is why *φ* is carried as an explicit coefficient, and the wood and value results are reported across its range (Section 2.7) rather than at the single value that fixed allocation implies. The correlations of Cabon et al. [23] suggest *φ* is well below unity, but a correlation between two time series is not the fraction of a flux that is realized, and we decline to convert one into the other. Nor do we adopt an intermediate value of *φ* as a central case: a value chosen from this evidence would be a second elicitation layered on the first, and the bound of Table 3 is the more defensible object. What is still missing is a water-potential threshold. Güney et al. [15] bound growth by climate, not by ψ, and no turgor or xylogenesis threshold has been published for *Cedrus libani* or *Juniperus excelsa*; establishing one—with the dendrometer and microcore methods already applied to these species in the Taurus—is the measurement that would convert the bound of Table 3 into an estimate.

The assimilation results are unaffected by any of this. The 34.1% reduction in potential GPP, its partition into an obligate and a drought-attributable component, and the gradient in that partition are statements about carbon uptake, and they stand whether or not the uptake would have become wood. Read in that order—a structural statement about assimilation, a conditional statement about wood—the analysis says something about afforestation carbon accounting that a fixed-allocation model cannot: the carbon and the timber arguments for these species rest on different evidence and should not be given the same weight.

### 3.4. Silvicultural Leverage at High Elevation

The decomposition analysis shows that, across the cedar–juniper estate, avoidable carbon loss is primarily attributable to stomatal-drought limitation rather than photorespiration, a pattern more pronounced than in coastal pines. This is because these species inhabit cool, high-elevation sites where photorespiration is reduced, as well as dry sites where water limitation is significant. Three management responses follow from the structure of the loss, with a caveat that applies to all of them: none has been shown here to recover any part of it, and the drought-attributable tier is the ceiling on what they could address, not an estimate of what they would. First, elevation-aware thinning. Reducing stand density on drier lower and interior margins decreases competition for soil water and raises midday xylem potential in the retained trees. Its net effect on stand carbon is nevertheless ambiguous and should not be presented as a straightforward recovery of the stomatal term: thinning removes photosynthetic leaf area directly, and at high elevation the opening of the canopy raises incident radiation at the soil surface and can increase soil evaporation, offsetting part of the gain in transpirable water. The defensible claim is narrower—that thinning improves the water status and drought resilience of the residual stand, with a short-term carbon cost and an uncertain medium-term carbon sign—and it is on resilience rather than on carbon recovery that targeted release in overstocked, degraded juniper stands is justified [2,31]. Second, provenance selection and assisted migration: aligning seed sources with projected future climates—for instance, deploying the most anisohydric, deep-rooting cedar provenances at arid margins and the most persistence-oriented juniper genotypes on the steppe front—could enhance realized assimilation without altering species composition [14,15]. Third, species pairing along the elevational belt where cedar and juniper co-occur may mitigate the summer carbon deficit across the landscape, as the two species display complementary vulnerabilities.

### 3.5. Climate Refugia and the Progression in the Absence of Action

The losses computed here are not static. Under CMIP5 and CMIP6 ensembles for the eastern Mediterranean, summer temperatures over Türkiye are projected to rise substantially and summer precipitation is projected to decline, with the largest changes concentrated in Central Anatolia and the Lakes District—precisely the zones where our juniper stomatal fractions are highest [32,33,34,35]. Both shifts amplify these mechanisms: photorespiration rises as Rubisco specificity falls with temperature [10,11], and stomatal limitation worsens as soil moisture declines and VPD increases [8,36]. The paradox is that the same warming that deepens the deficit also raises these species’ relative value: as the climate envelope of commercial pines retreats, cedar and juniper become the species of last resort on an expanding area of marginal ground, and their stands become climate refugia. The physiological penalty quantified here is therefore a growing, not a shrinking, quantity.

### 3.6. Consequences for Carbon Accounting and Afforestation Policy

The ~3.6 million Mg CO_2_ yr^−1^-equivalent shortfall in carbon uptake identified here is confined to the closed-canopy stands of two species on marginal land, yet it is large enough to matter for national accounting. It is simultaneously a risk, in that sequestration from high-elevation afforestation may fall short of planning assumptions that do not account for physiological constraints, and a possible opportunity, in that interventions that recovered any part of the drought-attributable tier—thinning, provenance matching, assisted migration—would also improve stand resilience; whether they recover any of it is not demonstrated here. Because cedar afforestation on karst already constitutes Türkiye’s largest such program, incorporating an eco-physiological discount into its carbon projections would improve the realism of national estimates at low cost. Türkiye already assesses forest carbon stocks for emissions-trading purposes [20], and integrates ecosystem-service accounting into national forest management planning [21]; an eco-physiological discount is a natural extension of both. These implications apply to the closed-canopy stands modeled here. The 1.47 million ha of open juniper woodland and the sparsely closed cedar lie outside the model (Section 4.1); their management is a question of restoration rather than of stand-level silviculture, and nothing here should be extrapolated to them.

### 3.7. Limitations

Seven limitations merit explicit mention. First, potential GPP under the counterfactual is computed using an RUE × APAR framework anchored in MOD17 values [17,37]; the truly unconstrained optimum cannot be directly observed. Second, the stress fractions (Section 4.2) are expert estimates calibrated to published ranges (two of them set to the index-implied values of Table S1) rather than direct measurements for these two species, and they constitute the single largest source of structural uncertainty in the study. Third, both species show large within-zone variability in soils, microclimate, and stand history that zone-level aggregation smooths over, and inventory data at sub-regional resolution remain limited for juniper. Fourth, the interaction between photorespiratory and stomatal losses becomes increasingly non-linear at extreme combined stress, and our first-order coupling term is an approximation. Fifth, the monetary translation is indicative only. Sixth, the relative weights of the nine zones are carried from the distribution literature rather than from inventory sub-totals; perturbing them by a multiplicative factor with a 20% standard deviation changed no Monte Carlo median or interval bound by more than two units in the last reported digit (Section 2.5). That is because the species totals are fixed to the inventory and the units within a species differ little in their fractions; the test does not address a systematic misplacement of a zone boundary. Seventh, the results refer to the 884,361 ha of normally closed stands; the 1.61 million ha of sparsely closed cedar and juniper is excluded and no extrapolation to it is made. The Monte Carlo analysis shows that the proportional loss is stable across 40,000 joint parameter draws, with stress fractions and the coupling coefficient as the only sources of spread, because potential radiation-use efficiency and absorbed radiation largely cancel in the ratio of fluxes that share them. It does not test whether those fractions are centered correctly: the model returns a positive loss for any positive stress fraction, so the sign of the result is a property of the structure rather than a finding. Because parameters were sampled independently, positive correlations among the biological stress terms under a warming climate might modestly widen these intervals—a refinement, alongside formal spatial error transmission, that we leave to future work [38].

## 4. Materials and Methods

### 4.1. Study System and Production Zones

**Figure 5:**
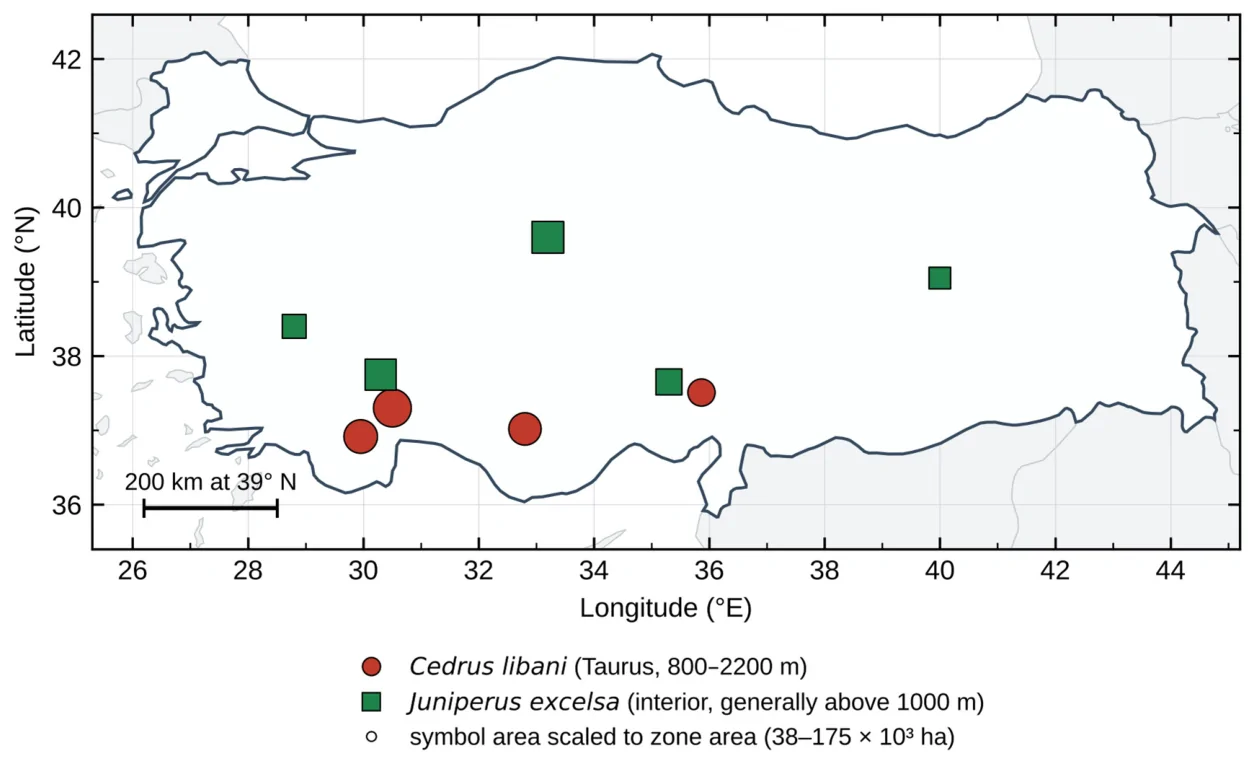
Distribution of *Cedrus libani* and *Juniperus excelsa* production zones in Türkiye, plotted on national and regional boundaries from the Natural Earth 1:50 m vector dataset in geographic (WGS84) coordinates. Symbols mark the centroid of each modeled zone × species unit and are scaled to its area (38–175 × 10^3^ ha); they denote zone position and extent, not mapped stand boundaries. Cedar (circles) occupies the Taurus arc at 800–2200 m; juniper (squares) occupies the drier interior and forest-steppe, generally above 1000 m. The stand and occurrence records at which the summer-stress index in Table S1 was evaluated lie within the units but do not generally coincide with these centroids.

The General Directorate of Forestry divides the national estate into ecological regions that differ in temperature seasonality, precipitation, and parent material [39]. We stratified the combined cedar–juniper range into nine zone × species units spanning the elevational and climatic envelope of the two species (Figure 5). *C. libani* was divided into four zones: the lower (800–1400 m) and upper (1400–2200 m) Western Taurus zones, the Central Taurus zone around Konya–Karaman, and the eastern Mediterranean massifs from the Aladağ and Kahramanmaraş ranges to the Amanos. Cedar is a montane species of the Taurus arc, forming closed stands on karstic limestone where annual precipitation ranges from about 400 to 1200 mm, and its natural regeneration on karst underpins Türkiye’s largest afforestation program [3,40,41].

*J. excelsa* was divided into five zones: the Lakes District and southwestern Anatolia, the Central Anatolian forest-steppe, the high Central and Eastern Taurus regions, the Aegean inner hinterland, and the high margins of Eastern Anatolia. Juniper occupies the drier, generally higher ground above about 1000 m, on stony limestone soils where precipitation ranges from roughly 250 to 1000 mm [42,43]. It is slow-growing, with annual stem increments roughly 60% lower than co-occurring cedar under matched conditions, but it grows about 15% more hours per year and survives drought at precipitation levels that exclude cedar [15,31]. Zone areas are given in Table 4 and their basis in the paragraph below; they total 505 × 10^3^ ha for cedar and 379 × 10^3^ ha for juniper.

Zone areas are taken from the 2025 national forest inventory [1], using the normally closed (*normal kapalı*) class only: 505,413 ha of *C. libani* and 378,948 ha of juniper, 884,361 ha in total. The sparsely closed (*boşluklu kapalı*) class—138,736 ha of cedar and 1,470,578 ha of juniper—is excluded because the absorbed-radiation and radiation-use-efficiency values used here describe closed stands and would overstate assimilation on open woodland of 10% crown cover or less. The national inventory does not resolve area by zone, so the relative weights of the nine units were retained from the published distribution literature and the species totals rescaled to the inventory figures. All estate-level results therefore refer to the closed-canopy cedar and juniper estate, not to the full extent of the two forest types.

### 4.2. Estimation of Zone-Specific Stress Fractions

Converging gas-exchange, dendrometer, and dendroclimatic evidence was used to assign each zone a fraction of potential GPP lost to photorespiration (*f*_PR_) and to drought-induced stomatal limitation (*f*_ST_), with the supporting source identified for every entry (Table 5). The resulting pairs of fractions and their position relative to lines of equal combined reduction are shown in Figure 2a. Because stomatal closure inflates photorespiration through the intercellular CO_2_ pathway, the two losses were treated multiplicatively, the photorespiratory fraction being amplified by a dimensionless coupling coefficient *λ*, so that the effective combined reduction is:

1 − (1 − *f*_PR_ ⋅ (1 + *λf*_ST_)) × (1 − *f*_ST_) (1)

The coupling coefficient *λ* is not a fitted constant. It is derived in the companion study for *Pinus brutia* and *Pinus nigra* [27] from the ratio of the CO_2_ compensation point (Γ*) to the chloroplastic CO_2_ concentration (Cc), which sets photorespiratory CO_2_ release per unit gross carboxylation. Stomatal closure raises that ratio by two multiplicative routes: a diffusive route, because assimilation becomes diffusion-limited and Cc must fall, and a thermal route, because the loss of latent cooling raises needle temperature and Γ* rises with it following the temperature functions of Bernacchi et al. [5]. At 28 °C with 20% stomatal loss, the diffusive route requires Cc to fall from 193 to 159 µmol mol^−1^ (an amplification of 1.21), and a 4 °C rise in needle temperature further amplifies Γ* by 1.22. Both routes are general properties of C_3_ leaves rather than of a species: the temperature response of Γ* is that of Rubisco [5], and the diffusive route follows from the fall in *C*_c_ that a given stomatal loss imposes. Their magnitudes were, however, evaluated for pine: the *C*_c_ response depends on the A–*C*_i_ relation of the species, and the 4 °C needle-temperature rise on leaf dimensions and boundary-layer conductance. What is elicited separately for cedar and juniper is the size of the stomatal loss, *f*_ST_. The transfer of λ to these species is therefore bounded rather than exact, which is why the derived envelope is carried through the uncertainty analysis and the sensitivity of the headline figures to λ is reported in Section 2.4. The amplification factor (1 + *λ f*_ST_) is the product of the two routes, 1.48; evaluated at the reference stomatal loss *f*_ST_ = 0.20, this gives *λ* = 2.40, within a derived envelope of 1.45–2.85. We adopt *λ* = 2.5, the value used in the companion study, and carry the full envelope through the uncertainty analysis of Section 4.6. Setting *λ* = 0, which removes the coupling entirely, is reported in Section 2.4 as a bounding case, together with the effect of the envelope on the headline figures.

The fractions were assigned by the same four-step structured elicitation used in the companion study [27]. First, process bounds were taken from the C_3_ gas-exchange literature. Second, a summer-stress index combining an elevation band, a maritime-buffering class, and the July–August soil-moisture minimum placed each zone within those bounds. Third, species-specific trajectories were applied, cedar and juniper differing in their photorespiratory and stomatal response to the same index. Fourth, the resulting rank order was checked against the dendroclimatic evidence. Each fraction carries a ±25% plausibility interval; it is an expert judgment of plausible width, applied uniformly to every zone and carried as a 2σ range in the uncertainty analysis, not a statistical confidence bound. Table S1 records, for every zone, the elevation band, the maritime-buffering class and the contextual source consulted, together with a reproducible evaluation of the summer-stress index from public data, so that the assignment can be followed and contested step by step. For that evaluation, the index was built from mean stand elevation, distance to the coast, and the July–August climatic water deficit of TerraClimate [44] over 2000–2023 at 35 published stand and occurrence records of the two species [40,45,46,47,48,49,50,51,52,53]. The water deficit replaces the soil-moisture minimum judged in the elicitation because it is available at stand scale from one public dataset and integrates atmospheric demand with soil-water supply. Mapping this index linearly onto the species bounds gives an index-implied value for each fraction; for 16 of the 18 elicited fractions the index-implied value lies inside the fraction’s ±25% plausibility interval, one at the limit at the reported precision. Because the species bounds are the extremes of the elicited values, the comparison tests the ordering and spacing of the units within a species, not the level of the fractions. The two elicited values whose index-implied counterparts fell outside that interval (the Central Taurus stomatal fraction, 20%, and the Central Anatolian photorespiratory fraction, 10%) were replaced by the index-implied values (14% and 13%; Table 5), so that for every fraction used the index-implied value lies inside its ±25% plausibility interval. Table S1 records both values and shows that, with the elicited values, the estate-wide reduction would be 34.9% instead of 34.1% of potential GPP and the obligate share would be 37% instead of 39%, within the Monte Carlo intervals of Table 2. Two things should be stated plainly. No zone has a direct measurement of either fraction for either species: the sources in Table 5 are contextual, establishing that a zone is warmer, drier, or more productive than its neighbors rather than measuring its photorespiratory or stomatal loss. And because the fractions are structured expert estimates, every result that follows is conditional on them; their consequences are evaluated explicitly in Section 2.4, Section 3.1 and Section 4.6.

### 4.3. Counterfactual Model Structure

**Figure 6:**
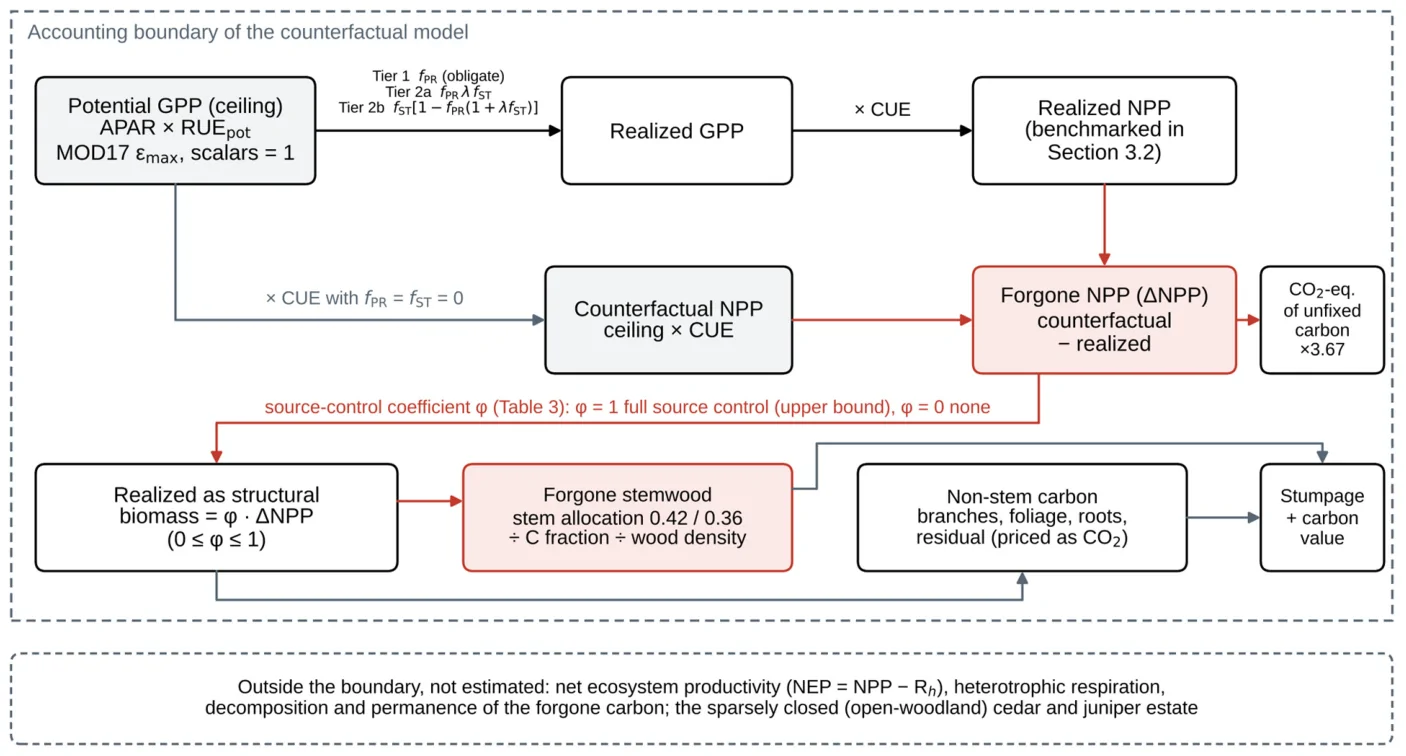
Carbon-accounting boundary of the counterfactual model. Quantities inside the dashed boundary are computed by Equations (1)–(4) and Section 4.5; the counterfactual ceiling is the MOD17 unstressed-scalar ceiling of Section 4.3. The wood branch is conditional on the source-control coefficient *φ* (Table 3). Net ecosystem productivity, heterotrophic respiration, decomposition and permanence of the forgone carbon, and the sparsely closed estate lie outside the boundary and are not estimated.

The counterfactual is defined by the ceiling against which it is computed, and that ceiling is not photorespiration-free. It is the radiation-limited productivity implied by the MOD17 maximum light-use efficiency for evergreen needleleaf forest with the temperature and vapor-pressure-deficit scalars set to unity—an empirically calibrated efficiency that already embodies Rubisco oxygenation at ambient CO_2_ and O_2_. The counterfactual therefore asks what the cedar and juniper estate would have produced from 2000 to 2023 at that unstressed-scalar ceiling, and the elicited photorespiratory and stomatal fractions measure the modeled distance from it rather than the carbon that abolishing photorespiration would release. In this construction, *f*_PR_ is the elicited photorespiratory loss as a fraction of potential GPP for the zone’s thermal regime (Section 4.2), applied to a ceiling that already embodies ambient photorespiration; the two biases this creates, one making the loss too small and one too large, are set out in Section 3.1. This scenario is not biologically feasible and serves only as an analytical tool to isolate the magnitude of these two constraints and determine an upper bound on potential gains from adaptive management. Figure 6 maps the quantities of the chain and the accounting boundary: the ceiling, realized GPP and NPP, forgone NPP and its stem and non-stem components, and the CO_2_-equivalent conversion lie inside it; net ecosystem productivity, heterotrophic respiration, decomposition and permanence lie outside it and are not estimated.

The model links three modules. First, a biophysical baseline computes NPP for each zone × species combination from absorbed photosynthetically active radiation (APAR), potential radiation-use efficiency (RUE_pot_), the photorespiratory and stomatal fractional losses, and a carbon-use efficiency (CUE) parameter. Second, a counterfactual NPP is obtained by relaxing both fractional losses to zero. Third, the NPP difference is converted into forgone stem volume using species-specific wood density and stem allocation, and, by the stoichiometric factor 3.67, into the CO_2_-equivalent of that forgone carbon. This conversion expresses modeled NPP carbon as CO_2_-equivalent; it does not estimate net ecosystem sequestration, which would require accounting for heterotrophic respiration, decomposition, and permanence. The biophysical baseline is:

NPP_actual_ = APAR × RUE_pot_ × (1 − *f*_PR_ ⋅ (1 + *λf*_ST_)) × (1 − *f*_ST_) × CUE (2)

The counterfactual relaxes both fractions,

NPP_cf_ = APAR × RUE_pot_ × CUE (3)

and the difference,

ΔNPP = NPP_cf_ − NPP_actual_ (4)

is the forgone carbon assimilation.

### 4.4. Parameterization

APAR values (780–1050 MJ m^−2^ yr^−1^) are lower than those for closed coastal pine canopies, indicating more open, higher-elevation stands and sparser juniper woodland canopies. Potential RUE (0.55–0.95 g C MJ^−1^) is anchored on the MOD17 evergreen-needleleaf value (0.962 g C MJ^−1^ [37]) and scaled downward for the intrinsically slow growth of these species, most steeply for juniper. Carbon-use efficiency was set at 0.45 (range 0.40–0.50), the value adopted for Mediterranean conifers in the companion study [27] and within the range reported for forest stands generally. Wood density was 0.53 Mg m^−3^ for cedar and 0.58 Mg m^−3^ for juniper, and stem allocation was 0.42 of total NPP for cedar and 0.36 for the shrubbier, multi-stemmed juniper. The cedar value derives from the component biomass tabulated by Durkaya et al. [54] for pure Taurus cedar stands, which gives a stem fraction of 0.52 of above-ground biomass; because that study measures no below-ground compartment, the figure was rescaled to a total-NPP basis using a below-ground fraction of 0.20. These values are specific to cedar and juniper and deliberately differ from the pine value used in the companion study. No equivalent partitioning study exists for *J. excelsa*, so its value is an assumption, and the juniper volume and value results are indicative for that reason. Using a standing-biomass stem fraction as the allocation of annual NPP assumes that carbon not fixed would have been partitioned as the accumulated biomass of these stands was. Two considerations make that an upper-end assumption rather than a central one. Across forest ecosystems, above-ground wood production takes 8–31% of GPP (10th–90th percentiles across sites), equivalent to roughly 18–69% of NPP at the carbon-use efficiency adopted here, and partitioning to wood increases with resource supply and is lower where water is limiting [55]. A stand-level biomass fraction, which integrates the favorable years in which most of the standing stemwood was laid down, therefore overstates the wood that additional carbon would form on the drier sites. The stem fractions adopted here (0.42, 0.36) lie within that range and must lie below its upper end, since stemwood is only part of above-ground wood production; any overstatement is absorbed by the source-control coefficient *φ*, which bounds the wood result from above (Section 4.5). Both are carried at ±15% through the uncertainty analysis, a wider band than the measurement alone implies, to absorb the below-ground correction. The resulting actual-NPP range is 1.11–2.83 Mg C ha^−1^ yr^−1^ across zones and 2.56–2.83 for cedar alone. The closest field figure for cedar, 1.6 Mg C ha^−1^ yr^−1^ [18], is a net sequestration rate rather than NPP and therefore sits below NPP by the heterotrophic respiration term; it bounds the model from below rather than validating its level, and the comparison with it and with regional MODIS forest NPP [17] is taken up in Section 3.2. Table 4 reports the central parameter set.

### 4.5. Translation to Stem Volume and Economic Value

Forgone carbon was converted to stem volume in three steps: the forgone NPP-C was multiplied by the species-specific stem allocation fraction, divided by a dry-biomass carbon fraction of 0.50 (Durkaya et al. [54] report 50.9% for cedar stemwood) to give forgone stem dry mass, and divided by species-specific wood density to give volume. The carbon fraction is held fixed and is not propagated in the uncertainty analysis. Forgone carbon was converted to CO_2_ using a factor of 3.67 times the forgone NPP-C. To contextualize the physiological deficit in socio-economic terms, the forgone volume was valued at a central stumpage price of US$110 m^−3^ for cedar and US$45 m^−3^ for juniper. Forgone CO_2_ was valued at a central price of US$30 Mg^−1^ CO_2_ for the annual figures, and only on the share of the forgone carbon that is not already valued as timber: the carbon held in the forgone stemwood is counted once, as stumpage, and the remaining carbon—branches, foliage, roots and the residual—is counted once, as CO_2_. The cumulative 2000–2023 net present value is not that annual figure repeated: the timber stream is carried back from 2023 on two assumed growth rates rather than observed series—real stumpage prices rising at 1.2% yr^−1^ and the affected area at 0.6% yr^−1^—so the cumulative figure is a back-cast scenario rather than a historical observation. The drawn terminal carbon price rescales the whole path, so carbon-price uncertainty is propagated through the net present value as well as through the annual figure; the carbon stream is valued on a price path rising linearly from US$5 Mg^−1^ CO_2_ in 2000 to US$30 in 2023 rather than at a flat present-day price, and both streams are then discounted to 2000 at a 4% real rate. Because the early years carry a much lower carbon price, the net present value is well below 24 times the current annual figure. This monetary valuation is provided for context only; the primary results are the physical carbon and volume quantities.

Both conversions presume that the carbon not fixed would have become biomass in the proportions observed in trees that did fix it. Because that presumption is exactly what the source–sink literature disputes, it is carried explicitly rather than silently. Let *φ* denote the fraction of the modeled forgone NPP that would in fact have been realized as additional structural biomass. *φ* = 1 is the source-limited case, in which allocation proceeds as observed and the conversions above apply unchanged; *φ* = 0 is the sink-limited case, in which the released carbon is respired, exuded or stored and no additional wood is formed. Every quantity downstream of NPP—forgone stem volume, retained CO_2_, stumpage value, carbon value and net present value—is linear in *φ*, so the results below are reported at *φ* = 1 and scale directly. No value of *φ* is adopted, because none has been published for any species; Section 3.3 sets out what the available evidence implies about its likely magnitude.

### 4.6. Sensitivity and Monte Carlo Uncertainty Analysis

Two complementary analyses were run. A one-at-a-time tornado analysis varied each input across its plausible range while holding the others at their central values. Then, to characterize the joint effect of parameter uncertainty, all inputs were propagated through a Monte Carlo simulation of N = 40,000 draws, following established uncertainty-propagation practice [38]. Each iteration drew independent values from distributions representing the plausible range of every uncertain parameter: the zone-specific photorespiratory and stomatal fractions, RUE_pot_, and APAR were varied by approximately ±25%, ±15%, and ±16% (2σ) about their central values using truncated-normal multipliers; CUE was drawn uniformly over 0.40–0.50; stem allocation and wood density were varied by ±15% and ±5%; and stumpage, carbon price, and the real discount rate were drawn from triangular distributions spanning the bounds given in Table 6. The choice of distribution follows the evidential basis of each parameter: truncated normals where a central value is supported by converging estimates and the bounds are symmetric (APAR, RUE_pot_, the two stress fractions, stem allocation, wood density), a uniform where the literature supports a range but no preferred value within it (CUE), and right-skewed triangulars where a modal value is well defined but the upper tail is long (stumpage, carbon price), or where bounds are set by policy convention (discount rate). The multipliers were truncated at the bounds given in Algorithm S1, and the fractions were additionally capped so that no draw could produce a negative flux or a fraction outside [0, 1]. The coupling coefficient *λ* was drawn over its derived envelope of 1.45–2.85 (Section 4.2). The full specification is given in Table 6. Medians and 2.5th–97.5th percentile (95%) conditional uncertainty intervals are reported. They describe the spread of the assigned parameter ranges, not the accuracy of the model. The draws were generated with NumPy’s default generator from the fixed seed 20260715; Algorithm S1 gives the sampling and aggregation steps in pseudocode, so that the propagation can be reproduced from Table 4, Table 5 and Table 6 without the code. Zone areas were held fixed in the main analysis because the species totals are set by the inventory; a supplementary run perturbed the relative weights of the nine units by a multiplicative normal factor with a 20% standard deviation (clipped at ±60%) and renormalized them to the inventory totals (Section 2.5, Table S2). Parameters were sampled independently; the consequences of that simplification are considered in Section 3.7.

## 5. Conclusions

This study provides the first integrated, literature-based scenario model of the carbon penalty imposed by photorespiration and drought-induced stomatal limitation on Türkiye’s two principal high-elevation conifers. The photorespiratory and stomatal fractions are structured expert estimates of 8–16% and 14–28% of potential GPP by zone; after coupling, photorespiration removes 13–23% and stomatal limitation 11–23%, with the drought-attributable component, which includes the photorespiration that stomatal closure amplifies, accounting for the majority of the loss. Under the multiplicative interaction, the two mechanisms suppress NPP by 26–44% across zones, a weighted-mean reduction of 34.1% (Monte Carlo median 33.6%, 95% conditional uncertainty interval 31.0–36.4%, which describes the spread of the elicited ranges rather than the accuracy of the model). Partitioning that reduction shows that 39–46% of it, depending on how the jointly caused interaction is assigned, is obligate photorespiration, which management cannot remove, and 54–61% is drought-attributable; both are conditional model results that follow from the elicited fractions, not measured quantities.

In absolute terms, conditional on the MOD17 ceiling (Section 3.1), this is a forgone CO_2_-equivalent carbon of ~3.6 million Mg yr^−1^ across 884,361 ha of closed-canopy cedar and juniper and, at *φ* = 1, a forgone potential stem volume of ~1.5 million m^3^ yr^−1^, together equivalent to roughly US$ 0.20 billion annually at prevailing prices, of which the drought-attributable component is about US$ 0.12 billion. These wood and value figures hold only if the carbon not fixed would have become biomass in the proportions observed; conifer radial growth stops at water potentials far less negative than those at which these species still assimilate, so they are reported across the full range of that assumption (Table 3) and fall to zero at its sink-limited end. The assimilation partition does not depend on it. Monte Carlo propagation indicates that these conclusions hold across the joint parameter ranges assigned, which is a statement about the assigned ranges and not about the accuracy of the model.

The central contribution is not the size of the loss, which follows from the elicited stress fractions rather than being inferred from them, but its partition: only the drought-attributable component—2.23 Mt CO_2_ yr^−1^ of the 3.65 Mt CO_2_ yr^−1^ headline—could be addressed by management to any degree, and that share rises as sites become drier. It is a ceiling, not a recovery estimate: whether thinning, provenance-matched assisted migration or any other intervention recovers part of it has not been demonstrated here. Elevation-aware thinning, provenance-matched assisted migration and an eco-physiological discount in afforestation carbon projections should therefore be assessed against the manageable fraction rather than against the total. Their importance is expected to increase as summers become hotter and drier, and as these forests persist as the primary vegetation on Türkiye’s marginal uplands.

## Figures and Tables

**Table 1:** Aggregate annual deficit, central estimates at *φ* = 1 (full source control, an upper bound; Section 2.7 gives the dependence on *φ*). Cedar stumpage US$ 110 m^−3^, juniper US$ 45 m^−3^, carbon US$ 30 Mg^−1^ CO_2_ applied to the non-stem carbon only, so that the carbon in the forgone stemwood is valued once, as timber. Juniper stem allocation and wood density are assumptions (Section 4.4); the juniper volume and value entries are indicative.

Quantity	*C. libani*	*J. excelsa*	Combined
Total area (10^3^ ha)	505	379	884
Annual ΔNPP (10^6^ Mg C yr^−1^)	0.67	0.33	0.99
Annual forgone stem volume (10^6^ m^3^ yr^−1^)	1.06	0.41	1.46
Annual forgone CO_2_ (10^6^ Mg CO_2_ yr^−1^)	2.45	1.20	3.65
Annual deficit, indicative (US$ billion)	0.16	0.04	0.20

**Table 2:** Monte Carlo medians and 95% conditional uncertainty intervals (CUI; 2.5th–97.5th percentiles) for the principal outputs, from N = 40,000 draws. The intervals describe the spread of the assigned parameter ranges (Section 4.6); they are not confidence intervals for the accuracy of the model.

Quantity	2.5%	Median	97.5%
Combined GPP reduction (%)	31.0	33.6	36.4
Obligate share of the loss (%)	36.1	39.5	43.1
Forgone NPP (10^6^ Mg C yr^−1^)	0.82	0.98	1.15
Forgone stem volume (10^6^ m^3^ yr^−1^)	1.16	1.43	1.76
Forgone CO_2_ (10^6^ Mg CO_2_ yr^−1^)	3.03	3.58	4.23
Annual deficit (US$ billion yr^−1^)	0.16	0.22	0.32
Cumulative 2000–2023 NPV (US$ billion)	1.72	2.40	3.31

**Table 3:** Forgone wood, retained carbon, and value as a function of the source-control coefficient *φ*, the fraction of the modeled forgone NPP that would have been realized as additional structural biomass. *φ* = 1 is the source-limited case assumed by fixed-allocation models and reported in Table 1 and Table 2; *φ* = 0 is the sink-limited case. All entries are linear in *φ*.

φ	Forgone NPP Realized (10^6^ Mg C yr^−1^)	Forgone Stem Volume (10^6^ m^3^ yr^−1^)	Retained CO_2_ (10^6^ Mg yr^−1^)	Annual Value (US$ Billion)
1.00	0.99	1.46	3.65	0.20
0.75	0.75	1.10	2.73	0.15
0.50	0.50	0.73	1.82	0.10
0.25	0.25	0.37	0.91	0.05
0.00	0.00	0.00	0.00	0.00

**Table 4:** Central-estimate parameter set for the counterfactual model.

Zone × Species	Area (10^3^ ha)	APAR (MJ m^−2^)	RUE_pot_ (g C MJ^−1^)	*f*_combined_	NPP_actual_ (Mg C ha^−1^)	NPP_cf_ (Mg C ha^−1^)
*C. libani*·W. Taurus lower	175	1050	0.95	0.37	2.83	4.49
*C. libani*·W. Taurus upper	132	900	0.85	0.26	2.56	3.44
*C. libani*·Central Taurus	121	1000	0.90	0.30	2.82	4.05
*C. libani*·E. Mediterranean	77	1050	0.88	0.35	2.71	4.16
*J. excelsa*·Lakes District	106	850	0.65	0.37	1.58	2.49
*J. excelsa*·C. Anatolia steppe	115	800	0.55	0.44	1.11	1.98
*J. excelsa*·C/E Taurus (high)	67	820	0.62	0.35	1.49	2.29
*J. excelsa*·Aegean hinterland	53	880	0.66	0.36	1.68	2.61
*J. excelsa*·E. Anatolia (high)	38	780	0.58	0.36	1.31	2.04

**Table 5:** Estimated proportional reduction in potential gross primary production attributable to photorespiration and drought-induced stomatal limitation, by zone and species.

Zone	Species	Photoresp. Loss (% GPP)	Stomatal Loss (% GPP)	Combined NPP Reduction (%)	Contextual Sources
W. Taurus lower (800–1400 m)	*C. libani*	16	18	37	Güney et al. [15]; Epron [12]
W. Taurus upper (1400–2200 m)	*C. libani*	10	14	26	Güney et al. [15,40]
Central Taurus (Konya–Karaman)	*C. libani*	14	14	30	Ladjal et al. [14]
E. Mediterranean massifs (Adana–Kahramanmaraş–Amanos)	*C. libani*	15	17	35	Boydak [3]
Lakes District/SW Anatolia	*J. excelsa*	12	22	37	Özkan et al. [43]; Güney et al. [15]
Central Anatolia forest-steppe	*J. excelsa*	13	28	44	Carus [31]; Fontaine et al. [42]
Central/Eastern Taurus (high)	*J. excelsa*	9	24	35	Güney et al. [15]
Aegean/inner hinterland	*J. excelsa*	13	20	36	Özkan et al. [43]
Eastern Anatolia (high margins)	*J. excelsa*	8	26	36	Fontaine et al. [42]

Combined NPP reduction = 1 − (1 − *f*_PR_ × (1 + *λ f*_ST_)) × (1 − *f*_ST_). The interaction term captures the inflation of photorespiratory loss by stomatal closure. The Central Taurus stomatal fraction (14%) and the Central Anatolian photorespiratory fraction (13%) are the index-implied values of Table S1 (Section 4.2); the elicited values were 20% and 10%.

**Table 6:** Parameter distributions used in the Monte Carlo uncertainty propagation (N = 40,000 draws). Truncation bounds are given in Algorithm S1. Zone-specific central values are given in Table 4 and Table 5.

Parameter	Distribution	Central Value	Range/Spread
APAR	Truncated normal	zone-specific	±16% (2σ)
RUE_pot_	Truncated normal	zone-specific	±15% (2σ)
*f*_PR_ (photorespiratory)	Truncated normal	zone-specific	±25% (2σ)
*f*_ST_ (stomatal)	Truncated normal	zone-specific	±25% (2σ)
*λ* (coupling coefficient)	Triangular	2.5	1.45–2.85 (derived envelope)
CUE	Uniform	0.45	0.40–0.50
Stem allocation	Truncated normal	0.42/0.36	±15%
Wood density	Truncated normal	0.53/0.58 Mg m^−3^	±5%
Cedar stumpage	Triangular (right-skewed)	US$ 110 m^−3^	80–150
Juniper stumpage	Triangular (right-skewed)	US$ 45 m^−3^	30–70
Carbon price	Triangular (right-skewed)	US$ 30 t^−1^ CO_2_	15–80
Real discount rate	Triangular	4%	3–5%

## Data Availability

No new measurements were collected; all inputs are published values, and Table 4, Table 5 and Table 6 provide the zone-level inputs, their sources, and the distributions used in the uncertainty propagation. The study does generate new model output—the deterministic zonal results, the 40,000-draw Monte Carlo sample and the derived economic series—but neither those outputs nor the code that produces them can be made publicly available: the counterfactual model is the subject of a pending patent application filed through the Kahramanmaraş Technology Transfer Office, and public disclosure would prejudice that application. The model is nevertheless fully specified in the text by Equations (1)–(4) together with the parameter values in Table 4, Table 5 and Table 6, which is sufficient to reproduce the deterministic results reported here; the Monte Carlo propagation is specified by Table 6, the fixed random seed given in Section 4.6 and the pseudocode of Algorithm S1, and its summary outputs are given in Table 2 and Table S2. The climatic inputs of Table S1 are taken from the public TerraClimate dataset [44], and the stand coordinates at which they were evaluated are from published site descriptions [40,45,46,47,48,49] and public data records [50,51,52,53]. Requests for access may be addressed to the corresponding authors and will be considered under a confidentiality agreement.

## Supplementary Materials

The following supporting information can be downloaded at: https://www.mdpi.com/article/10.3390/plants15213441/s1. Table S1: Derivation of the zonal photorespiratory and stomatal fractions (Section 4.2); Table S2: Monte Carlo medians and 95% conditional uncertainty intervals (2.5th–97.5th percentiles, N = 40,000) with zone areas fixed (main analysis, Table 2) and with the relative weights of the nine zone × species units perturbed by a multiplicative normal factor with a 20% standard deviation (clipped to 0.4–1.6) and renormalized within each species to the inventory totals (505,413 ha cedar, 378,948 ha juniper); Algorithm S1: Counterfactual model (Equations (1)–(4)) and Monte Carlo propagation, in pseudocode. References [3,5,7,12,14,15,31,40,42,43,44,45,46,47,48,49,50,51,52,53] are cited in the Supplementary Materials.
