# Supplementary material for: Photorespiration, Drought and the Assimilation–Growth Gap: Bounding the Forgone Wood in High-Elevation Cedrus libani and Juniperus excelsa Forests of the Eastern Mediterranean
Source: Plants (Basel). 2026 Nov 5;15(21):3441. doi: 10.3390/plants15213441 (PMC13765584; doi:10.3390/plants15213441)
Supplement: Supplementary file 1 [file plants-15-03441-s001.zip › plants-4676659-supplementary.pdf]

Supplementary Materials

Photorespiration, drought and the assimilation–growth gap: bounding the forgone wood in high-elevation *Cedrus libani* and *Juniperus excelsa* forests of the Eastern Mediterranean

Emre Yazar, Bülent Akgün, Emre Babur, Sinan Kordon and Erdi Ekren

Table S1. Derivation of the zonal photorespiratory and stomatal fractions (Section 4.2).

The fractions in Table 5 were assigned by the four-step structured elicitation of Section 4.2: literature process bounds (Step 1); placement of each zone within those bounds by a summer-stress index judged from its elevation band, a maritime-buffering class and the July–August soil-moisture minimum (Step 2); species-specific trajectories (Step 3); and a check of the resulting rank order against the dendroclimatic evidence cited in the last column (Step 4). The index columns below are a reproducible evaluation of that placement made after elicitation, using public data from published stand and occurrence records for each unit (note d): mean stand elevation, distance to the coast, and the July–August climatic water deficit from TerraClimate. The value each fraction would take by linear mapping of this index onto the species bounds (index-implied value) is reported next to the value assigned. Two elicited values lay outside their plausibility interval of the index and were replaced by the index-implied values in Table 5 and in all results; they are shown as "used (elicited)" and explained in note e. No zone has a direct measurement of either fraction for either species. Reference numbers follow the main text.

Zone (stands, note d)	Species	Elevation band (m) / mean stand elevation (n stands)	Maritime-buffering class (mean distance to coast)	Jul–Aug climatic water deficit, 2000–2023 (TerraClimate; mm); Jul–Aug precipitation (mm) for context	Summer-stress index: thermal axis T / hydric axis H (rank within species)	Index-implied value (linear mapping onto the species bounds) and comparison with the assigned value	fPR (%) used (elicited)	fST (%) used (elicited)	Contextual source
W. Taurus lower (800–1400 m)	*C. libani*	800–1400 / 1268 (n = 4)	1.5 (mean of stand classes) (29 km)	343 mm; precipitation 23 mm	T = +0.98 (1/4); H = -0.09 (2/4)	T rank 1/4 → f_{PR} 16% by linear mapping of T onto the species bound 10–16%; H rank 2/4 → f_{ST} 18% by linear mapping of H onto 14–20%. Both assigned values have the mapped value inside their ±25% plausibility interval.	16	18	Güney et al. [15]; Epron [12]
W. Taurus upper (1400–2200 m)	*C. libani*	1400–2200 / 1766 (n = 6)	2 -lee/intermediate (47 km)	324 mm; precipitation 25 mm	T = -1.20 (4/4); H = -0.29 (3/4)	T rank 4/4 → f_{PR} 10% by linear mapping of T onto the species bound 10–16%; H rank 3/4 → f_{ST} 17.5% by linear mapping of H onto 14–20%. The assigned f_{PR} falls within its ±25% plausibility interval; the mapped fST is at the upper limit of the elicited 14% interval (10.5–17.5%) at the reported precision.	10	14	Güney et al. [15,40]
Central Taurus (Konya-Karaman)	*C. libani*	1500–1750 (stands) / 1653 (n = 3)	1.3 (mean of stand classes) (75 km)	317 mm; precipitation 19 mm	T = -0.71 (3/4); H = -1.06 (4/4)	T rank 3/4 → f_{PR} 11% by linear mapping of T onto the species bound 10–16%; H rank 4/4 → f_{ST} 14% by linear mapping of H onto 14–20%. The mapped f_{ST} (14.0%) lies 1.0 point below the ±25% plausibility interval of the elicited 20% (15–25%). The index-	14	14 (20)	Ladjal et al. [14]

						implied value replaces the elicited value in Table 5 and in all results (note e).			
E. Mediterranean massifs (Adana–Kahramanmaraş–Amanos)	*C. libani*	1325–1550 (stands) / 1441 (n = 4)	2 -lee/intermediate (83 km)	341 mm; precipitation 13 mm	T = +0.22 (2/4); H = +0.25 (1/4)	T rank 2/4 → f_{PR} 14% by linear mapping of T onto the species bound 10–16%; H rank 1/4 → f_{ST} 20% by linear mapping of H onto 14–20%. Both assigned values have the mapped value inside their ±25% plausibility interval.	15	17	Boydak [3]
Lakes District / SW Anatolia	*J. excelsa*	1047–1862 (stands) / 1436 (n = 5)	1.6 (mean of stand classes) (58 km)	320 mm; precipitation 20 mm	T = +0.24 (3/5); H = -0.73 (4/5)	T rank 3/5 → f_{PR} 10% by linear mapping of T onto the species bound 8–13%; H rank 4/5 → f_{ST} 21% by linear mapping of H onto 20–28%. Both assigned values have the mapped value inside their ±25% plausibility interval.	12	22	Özkan et al. [43]; Güney et al. [15]
Central Anatolia forest-steppe	*J. excelsa*	1015–1260 (stands) / 1122 (n = 4)	2.8 (mean of stand classes) (191 km)	354 mm; precipitation 17 mm	T = +1.61 (1/5); H = +1.25 (2/5)	T rank 1/5 → f_{PR} 13% by linear mapping of T onto the species bound 8–13%; H rank 2/5 → f_{ST} 26% by linear mapping of H onto 20–28%. The mapped f_{PR} (13.0%) lies 0.5 points above the ±25% plausibility interval of the elicited 10% (7.5–12.5%). The index-implied value replaces the elicited value in Table 5 and in all results (note e).	13 (10)	28	Carus [31]; Fontaine et al. [42]
Central/Eastern Taurus (high)	*J. excelsa*	1470–1980 (stands) / 1744 (n = 4)	1 – seaward (45 km)	321 mm; precipitation 14 mm	T = -1.10 (5/5); H = -1.19 (5/5)	T rank 5/5 → f_{PR} 8% by linear mapping of T onto the species bound 8–13%; H rank 5/5 → f_{ST} 20% by linear mapping of H onto 20–28%. Both assigned values have the mapped value inside their ±25% plausibility interval.	9	24	Güney et al. [15]
Aegean / inner hinterland	*J. excelsa*	1123–1460 (stands) / 1254 (n = 3)	2-lee/intermediate (97 km)	332 mm; precipitation 27 mm	T = +1.03 (2/5); H = -0.02 (3/5)	T rank 2/5 → f_{PR} 12% by linear mapping of T onto the species bound 8–13%; H rank 3/5 → f_{ST} 23% by linear mapping of H onto 20–28%. Both assigned values have the mapped value inside their ±25% plausibility interval.	13	20	Özkan et al. [43]
Eastern Anatolia (high margins)	*J. excelsa*	1587–1880 (stands) / 1734 (n = 2)	3 – continental (219 km)	367 mm; precipitation 12 mm	T = -1.06 (4/5); H = +1.86 (1/5)	T rank 4/5 → f_{PR} 8% by linear mapping of T onto the species bound 8–13%; H rank 1/5 → f_{ST} 28% by linear mapping of H onto 20–28%. Both assigned values have the mapped value inside their ±25% plausibility interval.	8	26	Fontaine et al. [42]

Step 1 — process bounds. Photorespiratory fraction 8–16% of potential GPP: the ratio Γ^*/C_c, which sets photorespiratory CO_2 release per unit gross carboxylation, evaluated with the $\Gamma^*(T)$ function of Bernacchi et al. [5] at C_c = 200 μmol mol^{-1}, is 8% at 7 °C and 16% at 19 °C, the span of mean growing-season canopy temperatures across the estate, and 21% at 25 °C (the "fifth of gross assimilation" of Section 1 [7]). Stomatal fraction 14–28%: the elicited envelope, its lower bound for the coolest, wettest unit and its upper bound for the driest unit, consistent with the dominance of stomatal limitation in the drought response of *Cedrus* gas exchange [12,14] and with the soil-rewetting-dependent drought response of *J. excelsa* [15].

Step 2 — index. Post-hoc evaluation of the placement: thermal axis T = z(−mean stand elevation); hydric axis H = ½ z(D) + ½ z(M), where D is the mean July-plus-August climatic water deficit (potential minus actual evapotranspiration) over 2000–2023, the water-balance measure of unmet evaporative demand that integrates atmospheric demand with soil-water supply; M is the

maritime-buffering class and z denotes standardization across the nine units. Within each species each axis is mapped linearly onto the species bounds of Step 3 (axis minimum → lower bound, maximum → upper bound). Because those bounds are the extremes of the assigned values, the comparison tests the ordering and spacing of the units within a species, not the level of the fractions. Classes are assigned per stand from its great-circle distance to the Natural Earth 1:50 m coastline: 1 = seaward, ≤ 60 km and seaward of the main Taurus crest; 2 = lee or intermediate, 60–150 km, or ≤ 60 km but in an interior basin behind the coastal range (Elmalı, Bucak, Acıpayam basins); 3 = continental, > 150 km; the class entered in H is the mean of the stand classes.

Step 3 — species trajectories. The same index value is mapped onto species-specific bounds: *C. libani* f_{PR} 10–16%, f_{ST} 14–20%; *J. excelsa* f_{PR} 8–13%, f_{ST} 20–28%. Juniper has lower photorespiration and higher stomatal conductance because its units lie on the cooler, drier side of the estate and resume growth only after soil rewetting [15,31].

Step 4 — check. The rank order within each species was checked against the dendroclimatic evidence in the last column; any index-implied value outside the ±25% plausibility interval of the assigned value is stated in the comparison column.

Note e — departures. Two elicited fractions lay just outside the ±25% plausibility interval of the value mapped by the index: the Central Taurus stomatal fraction (20% elicited; 14% mapped, because the Ermenek and Gökyurt cedar stands at 1500–1750 m show the smallest July–August water deficit of the cedar units) and the Central Anatolian photorespiratory fraction (10% elicited; 13% mapped, because the Eskişehir and Kırıkkale juniper plots at 1000–1300 m are the lowest-lying juniper stands); the upper W. Taurus stomatal fraction (14% elicited; 17.5% mapped) sits at the limit and is retained. The two index-implied values replace the elicited values in Table 5 and in every result of the main text. With the elicited values, the deterministic estate-wide result would be a 34.9% instead of a 34.1% reduction of potential GPP, the obligate share 37% instead of 39%, the forgone stem volume 1.50 instead of 1.46 million m^3 yr^{-1} and the forgone CO_2 3.73 instead of 3.65 million Mg yr^{-1}, all inside the Monte Carlo intervals of Table 2; no conclusion of the paper depends on the choice.

Data. a TerraClimate [44], 1/24° monthly grids (def = climatic water deficit, used in the index; ppt, reported for context; soil and tmax were extracted at the Elmalı and tree-ring stands but are not used), aggregated series agg_terraclimate_<var>_1950_CurrentYear_GLOBE.nc on the Northwest Knowledge Network THREDDS server, point extraction at each stand, January 2000–December 2023; packed values unpacked with the stated scale_factor/add_offset. Stands closer than one grid cell share values. b Coastline: Natural Earth 1:50 m. c Each assigned fraction carries a ±25% plausibility interval, propagated in the Monte Carlo analysis (Section 4.6); the criterion for agreement is that the index-implied value falls inside the ±25% interval of the elicited value; under this criterion 16 of the 18 elicited fractions agree, one of them (upper W. Taurus f_{ST}, mapped 17.5%) at the limit at the reported precision, and the two that do not were replaced by the index-implied values (note e). d Stands (lat, lon, elevation; source): W. Taurus lower (800–1400 m): Elmalı CRF T4 (36.578 N, 29.969 E, 1055 m; Güney et al. 2015 [40], Table 1); Elmalı CRF T3 (36.578 N, 29.985 E, 1355 m; Güney et al. 2015 [40], Table 1); Aykırıçay seed stand (Finike–Kumluca) (36.450 N, 30.167 E, 1300 m; Demirci & Bilir 2001 [47], Table 1 (1′ resolution)); Arpacık seed stand (Fethiye) (36.817 N, 29.233 E, 1360 m; Demirci & Bilir 2001 [47], Table 1 (1′ resolution)). W. Taurus upper (1400–2200 m): Elmalı CRF T2 (36.585 N, 30.020 E, 1665 m; Güney et al. 2015 [40], Table 1); Elmalı CRF T1 (36.584 N, 30.031 E, 1960 m; Güney et al. 2015 [40], Table 1); Elmalı İşletmesi tree-ring site (36.670 N, 29.920 E, 1800 m; ITRDB TURK005 [50]); Katrandağı tree-ring site (Bucak) (37.380 N, 30.600 E, 1469 m; ITRDB TURK019 [50]); Bölükandız tree-ring site (Acıpayam) (37.250 N, 29.070 E, 1850 m; ITRDB TUR062 [50]); Çığlıkara seed stand (36.550 N, 29.800 E, 1850 m; Demirci & Bilir 2001 [47], Table 1 (1′ resolution)). Central Taurus (Konya-Karaman): Ermenek-1 seed stand (36.533 N, 32.767 E, 1750 m; Demirci & Bilir 2001 [47], Table 1 (1′ resolution)); Ermenek-2 seed stand (36.500 N, 32.733 E, 1710 m; Demirci & Bilir 2001 [47], Table 1 (1′ resolution)); Gökyurt relict stand (Meram, Konya) (37.817 N, 32.033 E, 1500 m; Demirci & Bilir 2001 [47], Table 1 (1′ resolution)). E. Mediterranean massifs (Adana–Kahramanmaraş–Amanos): Kahramanmaraş Elmadağ seed stand (Andırın) (37.617 N, 36.467 E, 1550 m; Demirci & Bilir 2001 [47], Table 1 (1′ resolution)); Çimen Dağı cedar–fir association (Kahramanmaraş) (37.448 N, 36.655 E, 1550 m; Varol & Tatlı 2001 [49] (1450–1650 m); summit coordinate from GeoNames [53]); Meydan Plateau L2 (Aladağ, Adana) (37.492 N, 35.374 E, 1340 m; Ezer & Kara 2013 [48], Table 1); Pozantı seed stand (Adana) (37.500 N, 34.950 E, 1325 m; Demirci & Bilir 2001 [47], Table 1 (1′ resolution)). The stands with published coordinates used for this unit lie in its Adana–Kahramanmaraş part; the Figure 5 symbol is placed at their centroid (37.51 N, 35.86 E). Lakes District / SW Anatolia: Göller tree-ring site (Antalya–Burdur) (37.080 N, 30.520 E, 1047 m; ITRDB TURK018 [50]); Su Batan tree-ring site (Bucak) (37.420 N, 30.300 E, 1862 m; ITRDB TURK014 [50]); Elmalı juniper tree-ring site (36.600 N, 30.020 E, 1853 m; ITRDB TURK016 [50]); Isparta-Senirkent population (38.087 N, 30.803 E, 1369 m; Evren & Kaya 2021 [45], Suppl. Inf. 1); Antalya-Termessos population (36.979 N, 30.459 E, 1050 m; Evren & Kaya 2021 [45], Suppl. Inf. 1). Central Anatolia forest-steppe: Alpu plots (Eskişehir) (39.914 N, 31.157 E, 1260 m; Kahveci & Arslan 2021 [46], Table 1 (1235–1286 m)); Seyitgazi plots (Eskişehir) (39.377 N, 31.039 E, 1125 m; Kahveci & Arslan 2021 [46], Table 1 (1047–1202 m)); Elmadağ plots (Kırıkkale) (39.792 N, 33.264 E, 1015 m; Kahveci & Arslan 2021 [46], Table 1 (1010–1021 m)); Yahşıhan plots (Kırıkkale) (39.794 N, 33.351 E, 1090 m; Kahveci & Arslan 2021 [46], Table 1 (980–1195 m)). Central/Eastern Taurus (high): Ananardıç tree-ring site (Bolkar) (37.270 N, 34.550 E, 1800 m; ITRDB TURK040 [50]); Arpa Alanı tree-ring site (Bolkar) (36.950 N, 34.080 E, 1980 m; ITRDB TUR059 [50]); Neşeli tree-ring site (Bolkar) (37.200 N, 34.470 E, 1725 m; ITRDB TURK042

[50]); Mersin-Tarsus population (S Bolkar) (37.117 N, 34.460 E, 1470 m; Evren & Kaya 2021 [45], Suppl. Inf. 1). Aegean / inner hinterland: Afyon-Sandıklı population (38.340 N, 30.130 E, 1123 m; Evren & Kaya 2021 [45], Suppl. Inf. 1); Denizli-Acıpayam population (37.195 N, 29.134 E, 1460 m; Evren & Kaya 2021 [45], Suppl. Inf. 1); Denizli-Beyağaç population (37.298 N, 28.972 E, 1180 m; Evren & Kaya 2021 [45], Suppl. Inf. 1). Eastern Anatolia (high margins): Tunceli-Ovacık occurrence (herbarium W) (39.344 N, 39.477 E, 1880 m; Herbarium W 2014-0017791 via JACQ [52]); Elazığ occurrence (BAYLU; labeled subsp. polycarpos) (38.703 N, 39.216 E, 1587 m; GBIF occurrence 3978736536 [51]).

Table S2. Monte Carlo medians and 95% conditional uncertainty intervals (2.5th–97.5th percentiles, N = 40,000) with zone areas fixed (main analysis, Table 2) and with the relative weights of the nine zone × species units perturbed by a multiplicative normal factor with a 20% standard deviation (clipped to 0.4–1.6) and renormalized within each species to the inventory totals (505,413 ha cedar, 378,948 ha juniper). The same random seed (20260715) was used for both runs.

Quantity	Zone areas fixed (Table 2)	Zone weights perturbed, σ = 20% (clipped 0.4–1.6)
Combined GPP reduction (%)	33.6 (31.0–36.4)	33.6 (30.9–36.6)
Obligate share of the loss (%)	39.5 (36.1–43.1)	39.5 (36.1–43.1)
Forgone NPP (10^6 Mg C yr^{-1})	0.98 (0.82–1.15)	0.98 (0.82–1.16)
Forgone stem volume (10^6 m^3 yr^{-1})	1.43 (1.16–1.76)	1.43 (1.15–1.78)
Forgone CO_2 (10^6 Mg CO_2 yr^{-1})	3.58 (3.03–4.23)	3.58 (3.01–4.25)
Annual deficit (US$ billion yr^{-1})	0.22 (0.16–0.32)	0.22 (0.16–0.32)
Cumulative 2000–2023 NPV (US$ billion)	2.40 (1.72–3.31)	2.39 (1.72–3.32)

Algorithm S1. Counterfactual model (Equations (1)–(4)) and Monte Carlo propagation, in pseudocode. Inputs are the zone parameters of Tables 4 and 5, the global parameters of Section 4.4 and the distributions of Table 6; no other input is used. The code itself is withheld under the pending patent application (Data Availability Statement).

```
Inputs (per zone z = 1…9): area A_z (10³ ha), APAR_z (MJ m⁻² yr⁻¹), RUE_pot,z (g C MJ⁻¹), f_PR,z, f_ST,z (Tables 4–5)
Global: λ = 2.5; CUE = 0.45; carbon fraction c_f = 0.50; stem allocation a_s = 0.42 (cedar) / 0.36 (juniper);
        wood density ρ_s = 0.53 / 0.58 Mg m⁻³; stumpage p_s = 110 / 45 US$ m⁻³; carbon price p_C = 30 US$ Mg⁻¹ CO₂;
        discount rate r = 0.04; source-control coefficient ϕ (reported at 1, 0.75, 0.5, 0.25, 0)

DETERMINISTIC RUN
for each zone z:
    G_z ← APAR_z × RUE_pot,z / 100                       # potential GPP, Mg C ha⁻¹ yr⁻¹ (the MOD17 unstressed-scalar ceiling)
    f_e,z ← f_PR,z × (1 + λ f_ST,z);   f_c,z ← 1 − (1 − f_e,z)(1 − f_ST,z)                     # Eq. (1)
    NPP_act,z ← G_z (1 − f_c,z) CUE;   NPP_cf,z ← G_z CUE;   ΔNPP_z ← NPP_cf,z − NPP_act,z        # Eqs (2)–(4)
```

```
 5      T1_z ← G_z f_PR,z;   T2a_z ← G_z f_PR,z λ f_ST,z;   T2b_z ← G_z f_ST,z (1 − f_e,z)           # tiers (Section 2.2)
 6  combined reduction ← Σ_z A_z G_z f_c,z / Σ_z A_z G_z
 7  obligate share ← Σ_z A_z T1_z / Σ_z A_z (T1_z + T2a_z + T2b_z)      # interaction T2a assigned to drought
 8  forgone NPP (Mt C yr⁻¹) ← Σ_z A_z ΔNPP_z × 10³ / 10⁶;   CO₂-equivalent of forgone NPP ← 3.67 × forgone NPP   # Sections 2.2, 2.3, 5; independent of ϕ
 9  forgone stem volume_z (m³) ← ϕ ΔNPP_z a_s / (c_f ρ_s) × A_z × 10³;   stem carbon_z ← ϕ ΔNPP_z a_s × A_z × 10³
10  retained CO₂ (Table 3) ← ϕ × 3.67 × forgone NPP;   non-stem CO₂ ← 3.67 × (ϕ × forgone NPP − Σ_z stem carbon_z)   # stem carbon priced as timber
11  annual value ← Σ_species p_s × volume_species + p_C × non-stem CO₂                      # linear in ϕ (Table 3)
11b drought-attributable value ← annual value × (1 − obligate share); the same factor applied, draw by draw, to the NPV of line 12 (Section 2.6)
12  NPV ← Σ_{y=2000}^{2023} [timber_y + carbon_y] (1 + r)^−(y−2000),  with timber_y = timber_2023 × 1.012^(y−2023) × 1.006^(y−2023)
        and carbon_y = non-stem CO₂ × ramp_y × (p_C / 30) × 1.006^(y−2023),  ramp linear from 5 (2000) to 30 (2023) US$ Mg⁻¹

MONTE CARLO PROPAGATION (N = 40,000; NumPy default_rng, seed 20260715)
13  for n = 1 … N:
14      draw multipliers m_APAR,z ~ TN(1, 0.08; 0.7, 1.3), m_RUE,z ~ TN(1, 0.075; 0.7, 1.3), m_PR,z, m_ST,z ~ TN(1, 0.125; 0.4, 1.7)
            independently for each zone (TN(μ, σ; lo, hi) = normal draw clipped to [lo, hi]; the bounds lie ≥ 3σ from the mean,
            so clipping and truncation are indistinguishable at the reported precision; ±16%, ±15%, ±25% at 2σ)
15      draw λ ~ Triangular(1.45, 2.5, 2.85); CUE ~ U(0.40, 0.50); m_alloc,species ~ TN(1, 0.075; 0.7, 1.3); m_dens,species ~ TN(1, 0.025; 0.9, 1.1)
16      draw p_s,cedar ~ Tri(80, 110, 150); p_s,juniper ~ Tri(30, 45, 70); p_C ~ Tri(15, 30, 80); r ~ Tri(0.03, 0.04, 0.05)
17      f_PR,z ← min(f_PR,z m_PR,z, 0.6);  f_ST,z ← min(f_ST,z m_ST,z, 0.6)                          # physical bounds
18      recompute lines 2–12 with the drawn values (zone areas fixed)
19  report median and 2.5th/97.5th percentiles of each output over the N draws (Table 2; Figure 4)

SUPPLEMENTARY RUN (Table S2): as above, with A_z ← A_z × TN(1, 0.20; 0.4, 1.6) at line 18 (a 20% standard deviation of the
    relative weight, clipped at ±60%), renormalized within each species
    so that Σ_z A_z over cedar zones = 505,413 ha and over juniper zones = 378,948 ha.

TORNADO (Figure 3): each input in turn set to the low and high value of its range (Table 6), all others at their central values;
    Figure 3 shows the six most influential inputs.
```
